# Integrating Antimicrobial Therapy with Host Immunity to Fight Drug-Resistant Infections: Classical vs. Adaptive Treatment

**DOI:** 10.1371/journal.pcbi.1004857

**Published:** 2016-04-14

**Authors:** Erida Gjini, Patricia H. Brito

**Affiliations:** 1 Instituto Gulbenkian de Ciência, Oeiras, Portugal; 2 Nova Medical School, Faculdade de Ciências Médicas, Universidade Nova de Lisboa, Lisbon, Portugal; University of California, Los Angeles, UNITED STATES

## Abstract

Antimicrobial resistance of infectious agents is a growing problem worldwide. To prevent the continuing selection and spread of drug resistance, rational design of antibiotic treatment is needed, and the question of aggressive vs. moderate therapies is currently heatedly debated. Host immunity is an important, but often-overlooked factor in the clearance of drug-resistant infections. In this work, we compare aggressive and moderate antibiotic treatment, accounting for host immunity effects. We use mathematical modelling of within-host infection dynamics to study the interplay between pathogen-dependent host immune responses and antibiotic treatment. We compare classical (fixed dose and duration) and adaptive (coupled to pathogen load) treatment regimes, exploring systematically infection outcomes such as time to clearance, immunopathology, host immunization, and selection of resistant bacteria. Our analysis and simulations uncover effective treatment strategies that promote synergy between the host immune system and the antimicrobial drug in clearing infection. Both in classical and adaptive treatment, we quantify how treatment timing and the strength of the immune response determine the success of moderate therapies. We explain key parameters and dimensions, where an adaptive regime differs from classical treatment, bringing new insight into the ongoing debate of resistance management. Emphasizing the sensitivity of treatment outcomes to the balance between external antibiotic intervention and endogenous natural defenses, our study calls for more empirical attention to host immunity processes.

## Author Summary

The evolution and spread of antimicrobial resistance is a major global problem, and a cause of substantial human mortality. As the discovery of new antibiotics does not follow the rate at which new resistances develop, a more judicial use of available drugs is needed. Here we develop a mathematical model of within-host infection dynamics that combines the effects of pathogen clearance by the host immune system and by the antibiotics. Computer simulations and mathematical analysis are used to evaluate treatment protocols in order to identify those that can restore patient health and limit the overall pathogen burden and selection of resistance. We focus our study on infections with pre-existing resistance, and explore two main treatment strategies: the classical treatment, characterized by fixed drug dose and treatment duration, and the adaptive treatment that closely follows infection outcomes and patient symptoms. Our results highlight treatment strategies that promote synergy between host immunity and the antimicrobial drug. This can be achieved by moderate treatments that combine appropriate timing, reduced drug dosage, and short treatment durations. Our model is developed for bacterial infections but our framework and findings may apply to other biological scenarios featuring drug resistance.

## Introduction

Overcoming antimicrobial resistance is currently considered an international medical priority [1, 2]. The evolution of drug resistance affects our ability to treat new infections as well as carry out hospital procedures that rely on the prophylactic use of antibiotics such as surgeries and organ transplants. Despite extensive research, antimicrobial alternatives to antibiotics, are not yet a practical solution over current therapies (reviewed in [3]). It is thus critical to evaluate different treatment strategies in order to understand how the various parameters involved in the prescription of antibiotics can influence the selection and spread of drug resistance. Optimization of antibiotic treatments to increase the effective life span of drugs, while reducing both the probability of resistance evolution and the adverse effects of treatments, is a key component of hospital antimicrobial stewardship programs [4], as well as a research priority in evolutionary epidemiology [5–7].

The problem of preventing the emergence of resistance is augmented with the problem of resistance management once it is already present in a population [8]. Often, by the time bacterial infections cause symptoms and treatment is initiated, the within-host bacterial load is large enough to harbour mutants that are resistant to the treating antibiotic [9]. More importantly, in hospital settings, resistant bacteria can already be acquired upon infection, requiring specialized therapeutic regimes [10–12].

Classical wisdom in drug-resistance management recommends that treatments should be as aggressive as possible, using the highest possible dose to ensure that the pathogen load is eliminated, and to prevent *de novo* evolution of resistance mutations [13]. These aggressive therapies have recently been questioned on the basis that the stronger the treatment applied, the stronger the selection favouring resistant pathogens, in particular in infections harbouring pre-existent resistance. This conventional protocol of hitting hard and hitting fast might be relevant for highly mutable pathogens such as HIV, but in cases where resistant strains are more likely to be acquired in the community such as in TB [8] the advantages of aggressive therapies are less obvious [14]. Alternative strategies could include more moderate treatments, or adaptive regimens where doses and treatment durations closely follow patient health [14–16].

Current empirical and theoretical evidence has examples to support both therapeutic strategies, as well as for a mixed compromise such as high dose and short treatments (reviewed by Kouyos et al. [15]). For instance, experimental studies using rodent malaria parasites in laboratory mice have shown that less aggressive chemotherapeutic regimens substantially reduce the probability of onward transmission of resistance without significant changes in host pathology [16]. In contrast, varying concentrations of vancomycin *in vitro*[17] and *in vivo* using a rabbit model [18] has confirmed the advantage of high dose aggressive treatment in controlling the resistant populations of *Staphylococcus aureus*. This multitude of results indicates that the problem of devising general practices for treatment is far from settled. Conceptual frameworks can help compare aggressive and moderate chemotherapy [15], but quantitative systematic analyses are also needed. The current challenge is to identify among the diverse potential treatment regimes, those that minimize selection for drug-resistance while not compromising patient health [14].

A general principle advocated to guide rational development of patient treatment guidelines is to impose no more selection than is absolutely necessary. For this, it is important to understand when rules like ‘hit hard and hit early’ should apply [13], and when more moderate treatment regimes would be more effective. Mathematical models play instrumental role in this endeavour. When focused on population level dynamics they can evaluate and guide antibiotic use regimens for hospitals [11, 19, 20] or wider communities [21], generally in endemic, but also in epidemic scenarios (e.g. antiviral usage [22, 23]). When modelling pathogen dynamics within host, mathematical approaches can outline the mechanisms of interaction and feedbacks among pathogen types, and quantify how this basic ecology is modulated by one drug [24] or multiple drugs [25].

An important, but often overlooked factor in the process of infection clearance and resistance management is host immunity. A strong immune response can substantially reduce the need for long treatments, as evidenced by some acute infections tending towards shorter drug treatments in hosts with intact immunity [26–28]. The interplay between host immunity and antimicrobial drugs has recently been incorporated into mathematical models of infection [29–31]. Previous work [29] has shown that the presence of an immune response can narrow down the mutant-selection window (MSW), defined as the range of drug concentrations for which the drug is strong enough to remove the sensitive population [32], but insufficient to remove the partially resistant pathogen population. Along similar lines, Ankomah and Levin, [31], using an explicit resource-based model for the interaction between pathogen and host immunity, have investigated infection scenarios, separating the effects of pathogen-dependent and pathogen-independent immune responses. Yet, a quantitative understanding of host immunity as a player in optimal treatment of resistant infections remains under-developed.

A series of studies have recently addressed the role of timing of antimicrobial use at the population level [22, 23]. By considering the indirect and direct effects of antimicrobial use, models have found that optimal timing for treatment at the population level is well into the course of an epidemic, where the indirect effects of delays usually result from minimizing the degree of overshoot, i.e. minimizing the number of cases beyond the number that would be needed to reach the epidemic threshold. There are parallels between transmission processes at the population level and pathogen growth dynamics at the within-host level, where timing effects of antimicrobial therapy have also been shown to be important [33].

In this article, we combine these two important concepts to study antimicrobial treatment of drug-resistant infections: i) we zoom further into host immunity processes, and ii) we analyze explicitly the role of treatment timing on the success or failure of antibiotic therapies. We consider a dynamic mathematical model that describes the interaction between the host’s immune system, pathogen density, and antimicrobial treatment in mixed infections of drug-sensitive and pre-existing drug-resistant pathogen strains. By analysing a diverse range of therapeutic scenarios, and especially focusing on treatment timing, we uncover critical consequences for infection dynamics and selection of resistance, before, during and after treatment.

We also compare in depth through a mechanistic approach, classical and adaptive treatment protocols, applied to the same infection. To facilitate insight into the driving factors of treatment efficacy, we simplify many aspects of host-pathogen interaction, focusing on key features. We examine their interplay with treatment parameters, and their final impact on infection outcomes, such as total immunopathology, time to clearance, pathogen burden, and overall resistance. Our framework formalizes and broadens up the question of what it means for a treatment to be optimal and how such optimality can be achieved in practice.

## Methods

### Mathematical model

The within-host model is designed to investigate the interplay between antibiotic treatment regimes and host immune response in acute drug-resistant infections. Our formulation is based on a previous within-host model of infection dynamics [33], but here we consider two pathogen phenotypes: those sensitive to the drug, *B*_*s*_, and those partly resistant *B*_*r*_. These are distinguished by their intrinsic growth rates (*r*_0_ and *r*_1_) and killing rates by antibiotic (*δ*_0_, *δ*_1_). We consider *c* = *r*_0_ − *r*_1_ ≥ 0 to be the fitness cost of resistance [34] and *a* = *δ*_1_/*δ*_0_, (0 ≤ *a* ≤ 1) to represent the fitness benefit of resistance, i.e. the factor by which antibiotic killing rate is reduced in the resistant sub-population.

The action of host immunity, is considered explicitly, in terms of naive antigen-specific precursor cells *N*, effector cells *E*, and memory cells *M*. We thus implicitly consider those infections that may have escaped the first barrier of innate immunity in the host [35]. The pathogen-dependent immune dynamics represents a typical CD8+ T-cell mediated immune response [36, 37], but also describes broadly key features of CD4+ cell responses [38, 39]. These are major players against intracellular bacterial pathogens, such as *Listeria monocytogenes*[40], and *Legionella pneumophila*[41, 42], but have also been implicated in *Haemophilus influenzae*[39, 43] and protective responses against pneumococcal bacteria [44]. In the interest of generality, we keep the detail of immune responses to a minimal level. Thus, the model is inevitably a simplification of the complex interaction between host immunity, bacteria, and antibiotics [45]. However, the underlying assumptions do capture crucial aspects of the expected immune responses in acute infections. These include induction, activation, proliferation, decay and memory formation, typically studied in greater empirical detail in virus-host interactions [46, 47]. Several mathematical aspects of our formulation feature in other theoretical models of infection [30, 31, 48].

Within-host dynamics for a mixed infection with a drug-sensitive (*B*_*s*_) and pre-existing partially resistant (*B*_*r*_) strain are described with the following set of ordinary differential equations:

$$\begin{array}{ccc} \frac{dB_{s}}{dt} & = & r_{0}B_{s}-dB_{s}I-\delta_{0}B_{s}\eta \left(t\right)A_{m} \end{array}$$ (1)

$$\begin{array}{ccc} \frac{dB_{r}}{dt} & = & r_{1}B_{r}-dB_{r}I-\delta_{1}B_{r}\eta \left(t\right)A_{m} \end{array}$$ (2)

$$\begin{array}{ccc} \frac{dN}{dt} & = & -\sigma N\frac{B}{k+B} \end{array}$$ (3)

$$\begin{array}{ccc} \frac{dE}{dt} & = & \left(2\sigma N+\sigma E\right)\frac{B}{k+B}-hE\left(1-\frac{B}{k+B}\right) \end{array}$$ (4)

$$\begin{array}{ccc} \frac{dM}{dt} & = & fEh\left(1-\frac{B}{k+B}\right), \end{array}$$ (5)

where *B*(*t*) = *B*_*s*_(*t*) + *B*_*r*_(*t*) is the total pathogen load at time *t*, and *I*(*t*) = *N*(*t*) + *E*(*t*) + *M*(*t*) is the total number of immune cells activated to clear the pathogen. Naive precursor cells (*N*) are stimulated to divide and differentiate into effector cells (*E*) in response to increasing pathogen density. Effector cells proliferate further upon antigen stimulation at rate *σ* as long as pathogen is still in circulation. As bacteria are cleared, the majority of effector cells undergo apoptosis at rate *h* per cell, except for a fraction *f* that differentiate into memory cells (*M*) that persist indefinitely. All three types of immune cells act to kill pathogen, but effector cells represent the dominant arm of the host immune defense, in particular in primary infection, which we focus on.

An important model assumption is that the killing rate *d* by lymphocytes is equal for both pathogen sub-types, regardless of their antimicrobial susceptibility. Another important assumption regards the immunity stimulation function. For immune stimulation by antigen, a monotonically increasing saturating function of pathogen density (Hill function with coefficient 1) is assumed, where the parameter *k* represents the half-saturation constant for stimulation of lymphocytes to divide and differentiate. We will hereafter refer to this parameter as the host immunity threshold. To reflect the discrete nature of the pathogen, we assume an extinction threshold, when pathogen density of either sub-population falls below a critical level *B*_*ext*_.

Since the model is primarily designed to describe acute infection, we do not include a limiting resource for pathogen growth [24], assuming main control via host immune responses. A detailed description of model parameters is given in Table 1. Although our simulations are based on a limited set of parameter values, likely to apply to a range of acute infections, the theoretical analysis that we provide alongside simulations enables extrapolation of our results to settings and numerical values departing from the ones considered here. As in another recent study [31], the exact parameter values used for simulations do not reflect any particular antibiotic-species combination.

**Table 1:** Model parameters and interpretation.

Symbol	Interpretation	Value	Range	Units	Reference
*r*_0_	Sensitive bacteria growth rate	3.3	1–8	(*day*^−1^)	[33, 49]
*r*_1_	Resistant bacteria growth rate	1.1	(≤ *r*_0_)	(*day*^−1^)	[34]
*d*	Pathogen killing rate by lymphocytes	1 × 10^−5^	10^−5^–10^−4^	*μl*/*cell*/*day*	[50, 33, 46]
*δ*_0_	Killing rate of sensitive bacteria by antimicrobial drug	1	Scaled	*l*/*mg*/*day*	
*δ*_1_	Killing rate of resistant bacteria by antimicrobial drug	*aδ*_0_	Scaled	*l*/*mg*/*day*	
*a*	Antimicrobial susceptibility factor of resistant bacteria	0.1	0 ≤ *a* ≤ 1	-	Varied
*A*_*m*_	Average antibiotic concentration	1–50	0.03–128	*mg*/*l*	Varied, [51]
*σ*	Maximum immune cell recruitment/proliferation rate	2	1.2–3	(*day*^−1^)	[36, 33, 52]
*k*	Pathogen density where immune response grows at half its maximum rate	1 × 10^5^	10^4^–10^5^	cell/*μ*l	[36, 33]
*h*	Maximum effector cell decay rate	0.35	0.1–0.8	(*day*^−1^)	[36, 33, 52]
*f*	Fraction of effectors converting to memory cells	0.1	0.05–0.1	-	[53, 33],
*B*_*s*_(0)/*B*_*r*_(0)	Initial bacterial density	10/2	≪ *k*	cell/*μ*l	Fixed [54]
*N*(0)	Initial precursor cell density	200	15–1500	cell/*μ*l	[55], [33]
*τ*_1_	Classical treatment onset (delay)	1–5	< *t*_*peak*_ (Eq 16)	day	Varied, [56]
*τ*_2_	Duration of classical treatment	7	3–15	day	Varied, [56]
Ω	Pathogen density causing symptoms in adaptive treatment	<, =, > *k*	10^3^–10^7^	cell/*μ*l	Varied
*B*_*ext*_	Pathogen extinction threshold	10^−1^	≤ 1	cell/*μ*l	Fixed

Although graphical illustrations in this study use these specific values, the additional analyses provided enable general extrapolation of model results to different parameter combinations that may apply in other settings.

### Antimicrobial treatment: Classical and adaptive

To model antimicrobial treatment we use an indicator function *η*(*t*), which represents the rate of antimicrobial uptake per unit of time. The dose of the drug deployed is denoted by *A*_*m*_. The case when treatment onset (*τ*_1_) and duration (*τ*_2_) are fixed from the start corresponds to a classical treatment. The case when drug uptake depends on bacterial density within host corresponds to an adaptive regime. For classical treatment, the rate of administration of antimicrobials is:

$$\begin{array}{c} \eta \left(t\right)=\left\{\begin{array}{cc} 1 & \text{if}\;\tau_{1}\leq t\leq \tau_{1}+\tau_{2}\\ 0 & \text{if}\;t<\tau_{1}\;\text{or}\;t>\tau_{1}+\tau_{2} \end{array}\right.. \end{array}$$ (6)

In the adaptive regime, treatment onset and duration are influenced by the bacterial dynamics in the infected host. Previous authors have considered tight coupling between adherence to drug and bacterial load [29]. In this study, similar to the study by Ankomah and Levin [31], we only consider the simplest form of adaptive treatment that uses a threshold for total pathogen load, Ω, above which the patient takes the drug, and below which the patient does not. The rate *η*(*t*) of antibiotic administration per unit of time, becomes a direct function of pathogen load *B* and the threshold Ω:

$$\begin{array}{c} \eta \left(t\right)=\left\{\begin{array}{c} 1,\;\text{if}\;B(t)\geq \Omega \\ 0,\;\text{if}\;B(t)<\Omega . \end{array}\right. \end{array}$$ (7)

Thus, over a given treatment window, the net average amount of drug taken by the host per unit of time in the adaptive case, may be less than the actual administered dose. This alternative model of antimicrobial delivery could mirror a ‘take when feeling bad, stop when feeling good’ approach, requiring necessarily reliable translation between symptoms and pathogen load.

Focusing on the net effect of the antibiotic on the bacterial population, which has been shown to be relatively insensitive to changes in the frequency of administration of the drug [31], we neglect the explicit pharmaco-dynamics of the antibiotic. Thus we model only the average rate of antibiotic-mediated pathogen killing (represented by the product *δ*_0_ *A*_*m*_ and *δ*_1_ *A*_*m*_, respectively for *B*_*s*_ and *B*_*r*_), which simplifies analysis.

Both in the classical and adaptive regime, we explore treatment onset at various times over infection, departing from previous studies that typically link treatment initiation to a fixed pathogen load or the peak bacterial density [30, 31]. Our formulation of treatment delay is inspired by two recent studies [23, 33]. Its generality enables a deeper understanding of the trade-off induced by antibiotic treatment between reduction in host pathology and immunization, in the new context of resistant infections.

### Infection summary measures for simulations

Assuming an extinction threshold when pathogen density of each subpopulation within host reaches *B*_*ext*_, we can compute *t*_*ext*_, the extinction time, or clearance time. Because we simulate treated and untreated infections only up to a finite time horizon *T*, usually set to 30 days, infection duration is thus defined as:

$$\begin{array}{c} \text{D}=min(T,t_{ext}) \end{array}$$ (8)

The total resistance burden over the entire infection is calculated as

$$\begin{array}{c} R_{tot}=\int_{0}^{D}B_{r}\left(t\right)dt. \end{array}$$ (9)

The total pathogen burden over infection is $B_{tot}=\overset{D}{\underset{0}{\int }}B(t)dt$, and final host immune memory is *M*(*D*). We also track the resulting immunopathology [33], which roughly reflects the cumulative damage to host health due to pathogen killing by cells of the immune response and associated inflammation [57]. For the total immunopathology accumulated up to time *t*, *H*(*t*), following [33], we define: $H(t)=\overset{t}{\underset{0}{\int }}dB(s)I(s)ds$. As the pathogen population grows and host immunity builds up, the cumulative immunopathology due to immune-mediated killing and inflammation also grows following the infection dynamics. Upon pathogen clearance (or at the end of a simulation) the immunopathology accumulated over infection reaches

$$\begin{array}{c} H_{tot}=\int_{0}^{D}dB\left(s\right)I\left(s\right)ds. \end{array}$$ (10)

We perform a systematic analysis of these infection outcomes, varying treatment regimes (classical and adaptive) and model parameters, such as the fitness cost and benefit of resistance, and host immunity characteristics. We use as reference for comparison summary measures from infections in which no treatment is used. All simulations are performed in Matlab^®^ R2011a.

## Results

### Dynamics in the absence of antibiotics

**Fig 1:**
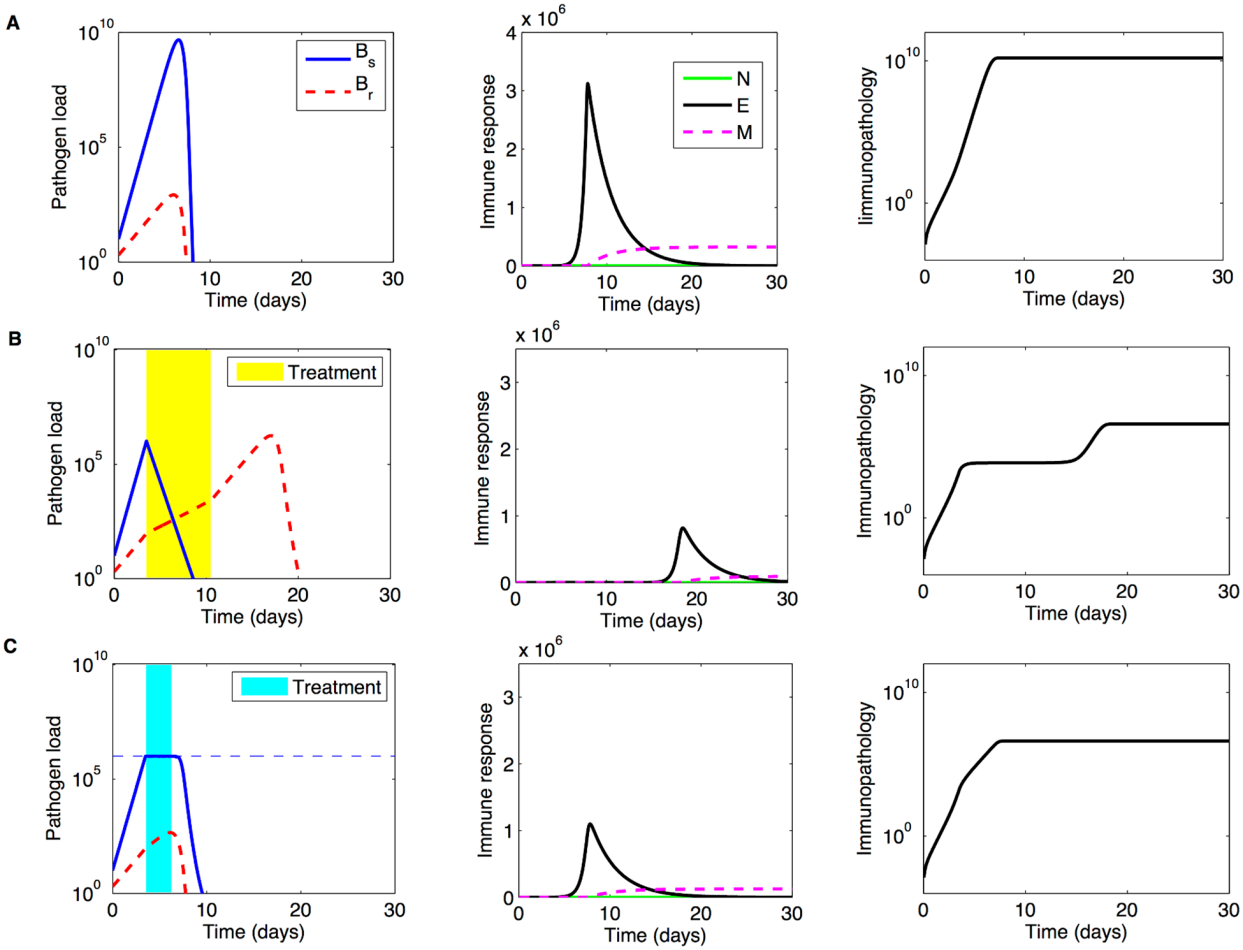
Illustration of model dynamics. **A)** Untreated infection. **B)** Classical treatment with: *A*_*m*_ = 6, *τ*_1_ = 3.5, *τ*_2_ = 7. **C)** Adaptive treatment with parameters Ω = 10^6^, *A*_*m*_ = 6. Other parameters as in Table 1. The resulting duration of adaptive treatment is 2.7 days. From the coupling to pathogen load, the effective rate of antibiotic uptake in adaptive treatment within such interval is 0.44, which implies a reduction in the effective dose from the prescribed *A*_*m*_ = 6 to about 3, sufficient to restrict growth of the drug-sensitive sub-population. The treatment onset *τ*_1_ = 3.5 days corresponds to Ω = 10^6^ (adaptive regime), where Ω > *k*.

In the absence of treatment, the infection follows a typical acute dynamics (Fig 1A). Sensitive bacteria grow initially quasi-exponentially, while immune responses are not yet active. Resistant bacteria also increase from their initially low numbers, but relatively more slowly, depending on their fitness cost *c* = *r*_0_ − *r*_1_. Resistant bacteria reach their peak around the same time as the sensitive sub-population, but at a lower density. As sufficient immunity gradually builds up during the bacterial growth phase, bacterial clearance is initiated, primarily through the action of effector cells. Following pathogen decline, effector cells also decline, with a fraction of them differentiating into persistent immune memory cells. High levels of acquired immune memory will act as pre-existing immunity in a secondary infection with the same pathogen and lead to rapid clearance. Mathematical analysis of the model in the absence of antibiotic confirms that stability of the infection-free state requires N* + M* > *max*(*r*_0_, *r*_1_)/*d* (see S1 Text, part I).

Below we derive analytical expressions, to understand how characteristics of the pathogen and of the host, represented by different parameters, interact to determine outcomes of infection. This serves as a starting point to then explore how perturbations like treatment, or variation in parameter values can affect these baseline dynamics. Focusing on the *‘expansion phase’* of immune dynamics, as in [48, 58], we can simplify the rates of change in total bacterial density and host immunity by the following sub-system:

$$\begin{array}{ccc} \frac{dB}{dt} & \approx & r_{0}B-dBI \end{array}$$ (11)

$$\begin{array}{ccc} \frac{dI}{dt} & \approx & \sigma I\frac{B}{k+B} \end{array}$$ (12)

where *I* = *N* + *E* + *M* and *B* = *B*_*s*_ + *B*_*r*_. By assuming negligible fitness cost of resistance (*r*_0_ ≈ *r*_1_), the equations above give somewhat an upper bound on total bacterial growth. Biologically, the relative magnitudes of various parameters satisfy: *B*_0_ ≪ *k*, *dI*_0_ ≪ *r*_0_, where *B*_0_ = *B*(0) is the initial pathogen density, and *I*_0_ = *I*(0) reflects the precursor frequency, i.e. the number of initial immune cells specific to the pathogen at the time of infection. Dividing the above equations, and integrating, we obtain:

$$\begin{array}{c} \text{log}\left(\frac{B+k}{B_{0}+k}\right)=\frac{r_{0}}{\sigma }\text{log}\left(\frac{I}{I_{0}}\right)-\frac{d}{\sigma }\left(I-I_{0}\right) \end{array}$$ (13)

This equation gives the relationship between the number of immune cells and parasite density at any given time during the bacterial growth phase. Thus, it allows us to calculate the level of immunity as a function of current pathogen load, and viceversa. Under this approximation, the peak pathogen load, in the absence of treatment, occurs when a critical level of host immunity has been reached, namely when

$$\begin{array}{c} I\approx I_{crit}=\frac{r_{0}}{d}. \end{array}$$ (14)

The peak pathogen density at the end of the growth phase in acute infection can be obtained from combining Eqs 13 and 14:

$$\begin{array}{c} B_{max}\approx \left(B_{0}+k\right){\left(\frac{r_{0}}{deI_{0}}\right)}^{r_{0}/\sigma } \end{array}$$ (15)

The time it takes for the pathogen load to reach its peak can be approximated considering two phases of growth: i) the time it takes the pathogen load to reach *k*, required for half-maximal immune stimulation, and ii) the time it takes the immune response subsequently to grow from its initial level to the critical level *I*_*crit*_. This dynamic decomposition focusing on *k* is an analytically convenient choice, yielding:

$$\begin{array}{ccc} t_{peak} & = & t_{k}+t_{k\to peak}\approx \frac{1}{r_{0}}\text{log}\left(\frac{k}{B_{0}}\right)+\frac{1}{\sigma }\text{log}\left(\frac{I_{crit}}{I_{0}}\right) \end{array}$$ (16)

Such expressions, taken together, convey how host immunity characteristics, e.g. initial immunity *I*(0) [55], or immune cell recruitment rate *σ*, affect different infection outcomes. These may vary with host age [59], or other aspects of immune competence. One can also notice above the importance of the host immunity threshold, *k*, and maximal pathogen growth rate, *r*_0_, which may vary too across host-pathogen systems. In the absence of the drug, the difference in growth rate between resistant and sensitive bacteria does not significantly affect the dynamics of immune build-up, peak bacterial load, or the cumulative immunopathology over infection. The cost of resistance (*c* = *r*_0_ − *r*_1_) only changes the relative frequency of resistance in the total pathogen load. This is because immunity gets equal stimulation from both bacterial types and kills them at the same rate.

After the immune *‘expansion phase’*, which leads to pathogen clearance, the *‘contraction’* and *‘memory’* phases of the immune response follow, provided that *h* > 0, described in detail by Eqs 3–5 of the full model. Summing those three equations, one can see that total immune response in the system keeps increasing whenever $B>\frac{kh(1-f)E}{\sigma (N+E)}$. This means some immune stimulation still continues during pathogen decline, thus the peak immune response reached over infection typically exceeds the critical value *I*_*crit*_ required for triggering clearance.

Notice that setting *h* = 0 in the full model would mimic a situation of non-waning immunity (at least non-waning in the time-scale of interest), quantitatively captured by the simple system of Eqs 11 and 12. Most of the analysis above could thus be useful to understand also such a scenario, where for instance, the final level of immunity *I*_*final*_ accumulated after infection could be calculated from Eq 13, by solving it for *B* = 0.

In all these theoretical scenarios, infection in principle resolves through action of host immunity, but depending on the severity of parameter values, the total damage to the host can be overwhelming, such that administration of drugs is required. By severity here we mean the clinical relevance or manifestation of *B*_*max*_ in the absence of treatment (Eq 15), e.g. how close this peak density would be to a pathogenesis or lethal threshold for the host [58]. This naturally depends on pathogen growth rate and host immune competence. For example, slowly-growing pathogens might never trigger symptoms in their host (thus may never need antibiotic treatment), and eventually will be cleared by the immune system without causing high levels of pathology.

Next, we analyze the full model with treatment, where antibiotics interact with host immunity. In treated infections, the presence of a drug-resistant pathogen sub-population becomes relevant in either regime of drug delivery (see Fig 1B and 1C).

### Dynamics with classical antibiotic treatment

**Fig 2:**
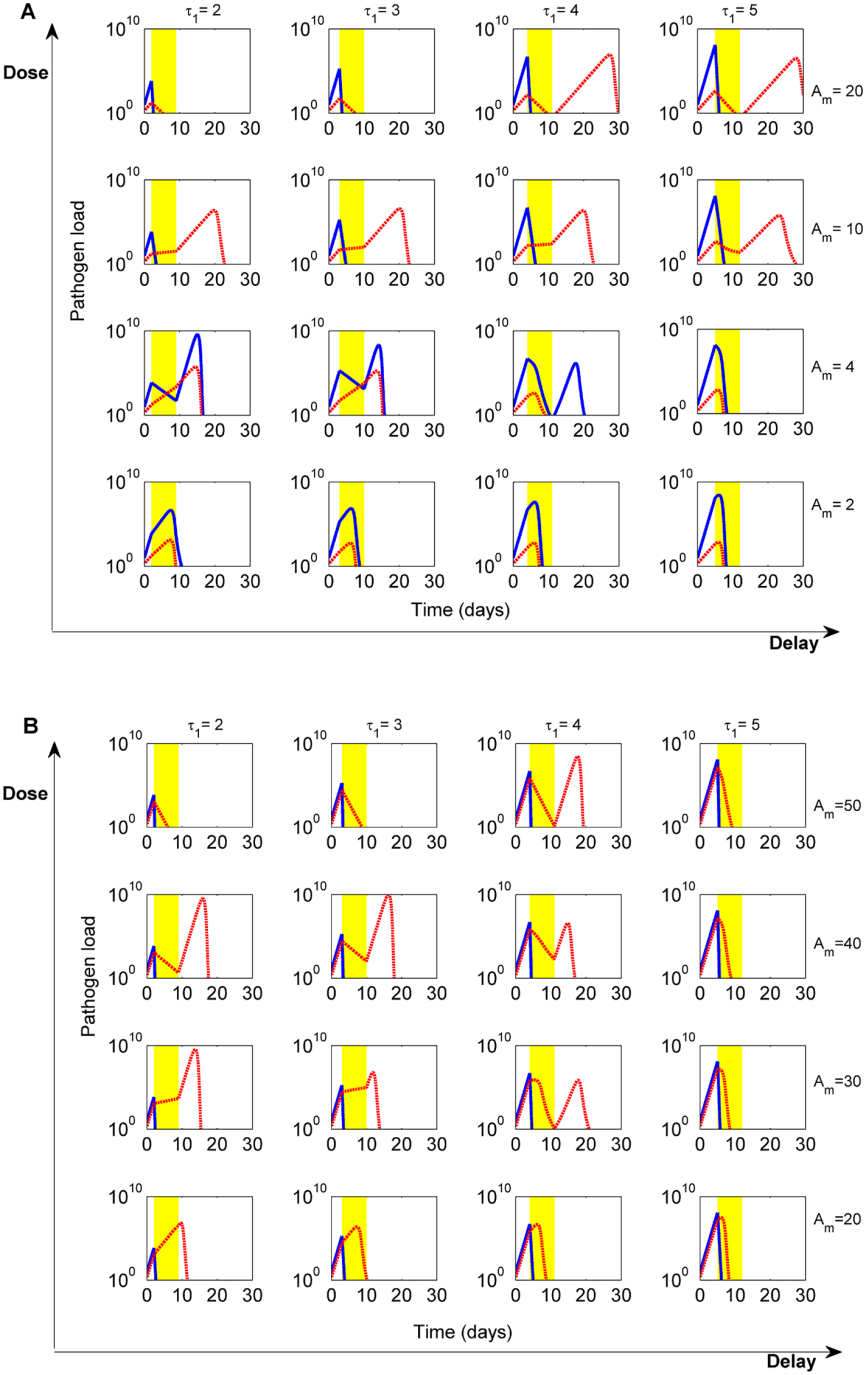
Dose-delay interaction during classical treatment with fixed dose and duration. Pathogen dynamics for the sensitive (blue) and resistant sub-population (red dashed line) are shown across different parameter combinations. **A)** High cost of resistance, *c* = 2.2. **B)** Low cost of resistance *c* = 0.1. Parameters as in Table 1, (*r*_0_ = 3.3) with treatment duration set to 7 days. Varying the cost of resistance, from A to B, shifts to higher values the range of critical doses that promote selection of resistance, from doses in [3.3, 11] to [3.3, 32]. However, we again observe an effect of bacterial clearance via synergistic interaction between drug and host immunity, when moderate doses are applied at appropriate times over the course of infection (here at *τ*_1_ = 4 days). All scenarios depicted achieved infection clearance (*B* < *B*_*ext*_) by 30 days, except in A) scenarios: *τ*_1_ = 4, 5, *A*_*m*_ = 20 and *τ*_1_ = 5, *A*_*m*_ = 10, where clearance occurred by 30.1, 31.2 and 30.6 days respectively. The minimum time to clearance is observed for smallest delay and highest dose in both A) and B), at 7.8 and 7.2 days post infection.

**Fig 3:**
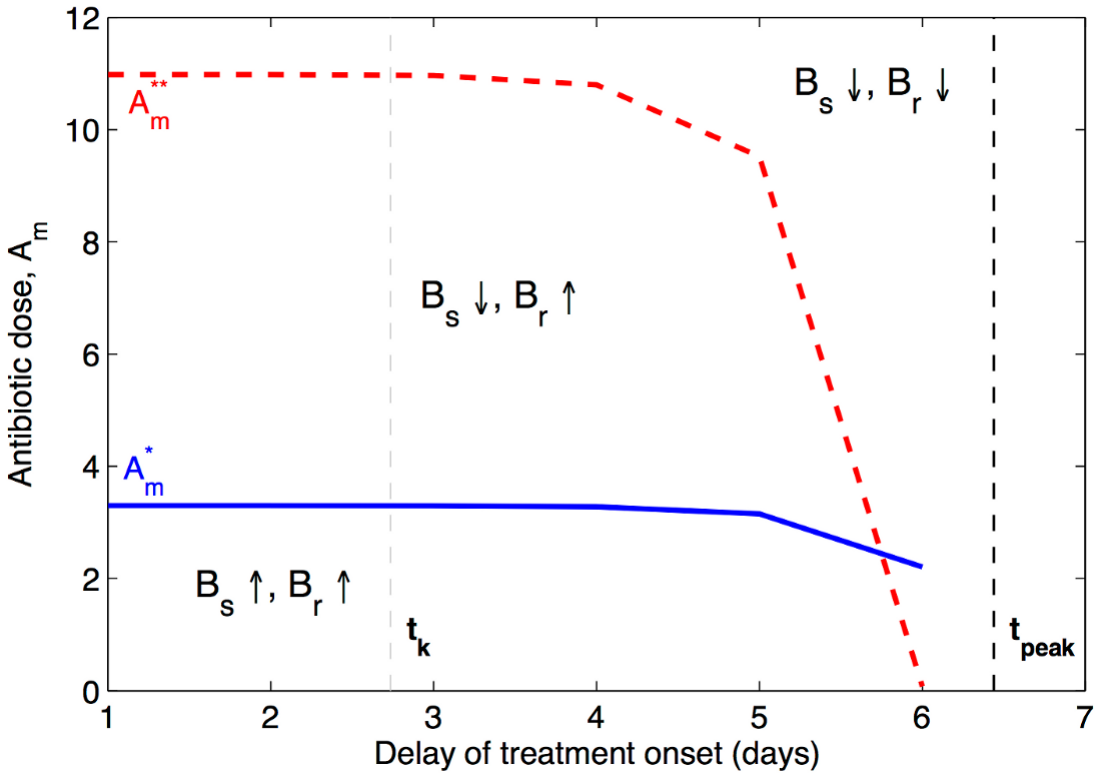
The critical antibiotic dose range, for bacterial behaviour immediately after treatment onset. As the treatment onset is postponed, and more immunity accumulates in the host, smaller doses can be used to interrupt the growth of each bacterial sub-population (blue line for *B*_*s*_, and red line for *B*_*r*_). Depending on how the actual dose, *A*_*m*_, that is deployed, sits in this range, different dynamic scenarios may ensue during treatment, with specific consequences for immune dynamics. In the lower dose range, immunity can still increase during treatment and assist in infection clearance. In the higher dose range, immune build-up is disrupted by treatment, and clearance can be achieved only by prolonged antibiotic pressure. Parameters as in Table 1. The time it takes the pathogen load to trigger immune activation (i.e. reach the immunity threshold *k*), in the absence of treatment, denoted by *t*_*k*_, is given by the gray vertical line. The time it takes the host immunity to trigger bacterial decline (i.e. time for *B*(*t*) to reach its peak), in the absence of treatment, denoted by *t*_*peak*_ is given by the black vertical line. An optimal delay usually sits in the middle of this range.

The effect of the antibiotic can be encapsulated as a reduction in the intrinsic per capita net growth rate of the two bacterial types during the treatment phase (*τ*_1_ ≤ *t* ≤ *τ*_1_ + *τ*_2_). The antibiotic reduces pathogen load and immunopathology, relieving the burden on host immunity (Fig 1B). However, its timing, dose, and duration can produce a diverse range of outcomes, as shown in Fig 2. With very aggressive treatments, resistant bacteria are not selected, and infections get cleared rapidly. In other cases, treatment cessation may result in a second infection peak, or even multiple peaks of bacteria, which may be equal to or even higher than pre-treatment levels, and consist of sensitive or resistant organisms. Treatment consequences vary especially depending on the phase of the infection in which treatment begins, where the growth potential of both strains is modulated by host immunity. To understand critical treatment parameters, we must consider the respective growth rates of bacterial subpopulations within host at the time *τ*_1_ when treatment is applied. In the presence of an immune response, the doses needed to halt growth of either subpopulation are decreasing functions of the immunity level *I*(*τ*_1_) upon treatment onset:

$$\begin{array}{c} A_{m}^{{}^{\prime }}\left(I\right)=\frac{r_{0}-dI}{\delta_{0}}\;\;\text{and}\;\;A_{m}^{{}^{\prime \prime }}\left(I\right)=\frac{r_{1}-dI}{a\delta_{0}}, \end{array}$$ (17)

for the sensitive and resistant strains respectively. In the absence of any immunity, the antibiotic doses that inhibit growth of sensitive and resistant bacteria are given by the maximum values:

$$\begin{array}{c} A_{m}^{*}=\frac{r_{0}}{\delta_{0}}\;\;\text{and}\;\;A_{m}^{**}=\frac{r_{1}}{a\delta_{0}}, \end{array}$$ (18)

where $A_{m}^{*}\leq A_{m}^{**}$, if the cost and benefit of resistance balance in such a way that *r*_0_ ≤ *r*_1_/*a* (the scenario we consider here). In general, depending on the level of immunity, thus on the delay for treatment initiation, either bacterial type can decline, persist or grow during treatment, subject to how the actual dose that is deployed, *A*_*m*_, sits in this critical range (Fig 3). As a consequence, immunity can also decline, persist or grow while antibiotics are applied. If during treatment, the net change in dynamics results in an excessive decline of pathogen-dependent immunity, there is a window of possibility for pathogen relapse after treatment cessation, in case complete clearance has not been achieved with the drug. The sub-population surviving at an advantage at the end of treatment, may be the one to dominate the relapse, provided that such advantage in total numbers is greater than its relative fitness cost in the absence of treatment (Fig 2A, *τ*_1_ = 2, *A*_*m*_ = 4). When such recrudescence is caused by resistant bacteria (e.g. for *τ*_1_ = 2, *A*_*m*_ = 10 in Fig 2A, or *A*_*m*_ = 30 in Fig 2B), the lower the fitness cost of resistance is, the faster the new peak will be reached after therapy stops. Clearly, the amount of drug interference with normal immune build-up during treatment depends on its dose and duration.

Thus, under pathogen density-dependent immunity, if bacteria persist or grow slightly during treatment it may not be so bad, given that such growth helps stimulate more immunity, and reduces the risk of relapse at the end of treatment. By the same argument, removal of antigen stimulus too rapidly during treatment may have adverse effects, because surviving pathogens at the end of therapy could re-grow if immune responses in the meantime have declined to subcritical levels (assuming waning immunity, *h* > 0). Selecting a moderate regime to balance between these scenarios is a challenge.

### Infection clearance through aggressive treatment and no involvement of the immune system

While finding an optimal intermediate regime, involving some degree of immune control, is far from trivial, the extreme therapeutic option that does not require immunity at all, is much easier to analyze. Such antibiotic treatment is bound to be of an aggressive type. Consider the total bacterial load at treatment onset *B*(*τ*_1_). The scenario of drug-only-mediated clearance can be represented as an exponential decay of both bacterial subpopulations during treatment. Notice that resistant bacteria are killed at lowest rate by the drug, so by approximating the total population decline at that lower rate, we explore the worst case scenario for the host. Resistant bacteria are also more likely to suffer a fitness cost (*r*_1_ ≤ *r*_0_), thus by approximating total population growth at its highest possible rate, *r*_0_, we are also considering a worst case scenario for the host. In this way, by being conservative in bacterial growth and decline during treatment, we obtain a sufficient criterion for ultimate clearance during classical treatment with dose *A*_*m*_ and duration *τ*_2_ as $B(\tau_{1})e^{(r_{0}-a\delta_{0}A_{m})\tau_{2}}\leq B_{ext},$ which is equivalent to requiring:

$$\begin{array}{c} A_{m}\geq \frac{1}{a\delta_{0}}\left[r_{0}-\frac{1}{\tau_{2}}\text{log}\left(\frac{B_{ext}}{B\left(\tau_{1}\right)}\right)\right] \end{array}$$ (19)

**Fig 4:**
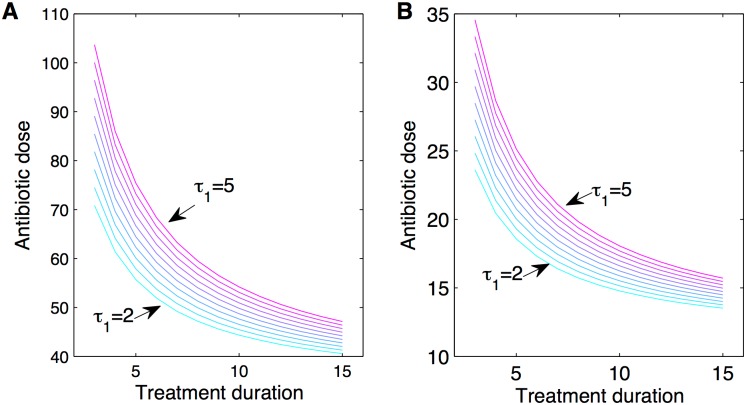
Aggressive treatment guaranteeing infection clearance using a classical regime, without relying on host immunity. Antibiotic dose and treatment duration can be traded-off against one another, at any treatment onset (lines depict *τ*_1_ varying between 2 days and 5 days), to achieve the same final result: infection clearance by the end of therapy. We apply Eq 19 to two infection scenarios: **A)** *a* = 0.1, very high resistance; and **B)** *a* = 0.3 lower resistance of the drug-resistant bacterial sub-population. Parameters as in Table 1.

Thus, if the dose and duration of classical treatment, in combination satisfy the above inequality, relative to the pathogen density at treatment onset *B*(*τ*_1_), and pathogen extinction threshold *B*_*ext*_, infection clearance by the end of treatment is guaranteed, without relying on host immunity. As the above expression shows, the earlier treatment begins, thus the lower *B*(*τ*_1_), the easier it is to meet the criterion with smaller doses and shorter treatment duration (Fig 4). Generally, the dose *A*_*m*_ and duration *τ*_2_, can be traded-off against one another, and still satisfy the clearance criterion for different pathogen loads at classical treatment onset. The caveat is to know whether these effects are possible with antibiotic doses below the toxic threshold for the patient. Notice, that the criterion in Eq 19, does not depend on the cost of resistance, and also does not exclude that clearance may be achieved with lower doses, because the additional pathogen killing by immunity, accumulated up to and during treatment, is not accounted for by this formula.

### Drug-immunity interplay and the role of host’s natural defenses

Here, we explore the interplay between antibiotic treatment and host immunity in the full dose range through numerical simulations of the complete model. In several ‘theoretical experiments’ (Fig 2), we vary treatment onset *τ*_1_, between 2 and 5 days post-infection, and consider treatment duration between 3 and 15 days, realistic for bacterial infections [56]. Such duration may correspond to the prescribed therapy by a doctor, or may reflect the actual adherence by the patient. Similarly, the delay can reflect the time over a typical infection course when a patient seeks treatment, and this may fluctuate from person to person.

Varying the antimicrobial dose, we observe that doses of the drug below $A_{m}^{*}$ (Eq 18) administered somewhat later over infection can be efficient in reducing the bacterial burden without promoting selection for resistance, because they yield the preponderate role in eliminating bacteria to the immune system. As soon as doses go above $\frac{r_{0}-r_{1}}{\delta (1-a)}$, the fitness differential between sensitive and drug-resistant bacteria is reversed (e.g. Fig 2A: *A*_*m*_ = 4, *τ*_1_ = 2). Small doses, just above $A_{m}^{*}$, start to interfere with immune build-up, but this interference decreases when treatment onset is delayed (moving along the delay axis in Fig 2A and 2B). Higher intermediate doses of the drug, between $A_{m}^{*}$ and $A_{m}^{**}$, instead, promote more selection of resistant bacteria during and after treatment, and ultimately infection clearance is achieved by the delayed action of the immune system (e.g. Fig 2A: *A*_*m*_ = 10). Yet, also here, optimal intermediate delays for initiating treatment, can help reduce host immunopathology and selection of resistance (S1 Fig). In contrast, higher doses of antimicrobial drug, beyond $A_{m}^{**}$, are able to induce immediately the decline of both sensitive and resistant populations (e.g. Fig 2A: *A*_*m*_ = 20, and Fig 2B *A*_*m*_ = 40), but at the risk of a resistant relapse if they are not high enough, or applied sufficiently long (Eq 19). At the extreme case of very aggressive treatment, the host experiences minimal immunopathology from infection, but also does not accumulate any immune memory.

As a result of interference by the drug, at certain intermediate doses, relapses in pathogen load can be maintained indefinitely. These arise when immunity at the end of treatment consists approximately only of effector cells *I* ≈ *E*, and coincides with *r*_0_/*d*, while pathogen load coincides with *B*(*t*) = *hk*/*σ* (see S1 Text, part I). Since these values are sufficient to yield *dE*/*dt* = 0 and *dB*/*dt* = 0, a persistence quasi-steady state is observed with oscillatory dynamics, as reported also in the model by [33]. Such oscillations typically arise in predator-prey systems, making it hard for the immune response to clear the pathogen in the short term. The total pathogen density may consist of sensitive or resistant bacteria, if the dose has been low or sufficiently high respectively. Given enough time however, conversion of effectors into memory cells will gradually build up enough persistent immunity to enable final clearance.

When fixing the delay *τ*_1_ for treatment onset, we find many dose-duration combinations that select for the same amount of resistance overall, although they lead to varying immunopathology (S2 Fig). On the other hand, many dose-duration combinations lead to the same immunopathology (clinically neutral range) [15], while corresponding to different bacterial burdens and infection duration. Testing for the advantages of increasing treatment duration, we find that these apply only in those combinations of dose and delay where treatment immediately induces net pathogen decline. In dose-delay combinations where treatment allows slight pathogen growth, thus co-stimulation of the immune system, we observe that increasing treatment duration does not significantly improve infection outcomes, as ultimate clearance is driven by host immunity. In cases where antibiotic doses just about prevent growth of *B*(*t*), keeping bacterial density at a too low level relative to the immunity threshold *k*, longer treatments may worsen outcomes: by delaying the relapse bound to occur at the end of therapy, and by increasing resistance selection.

### Numerical search for optimal classical treatment

**Fig 5:**
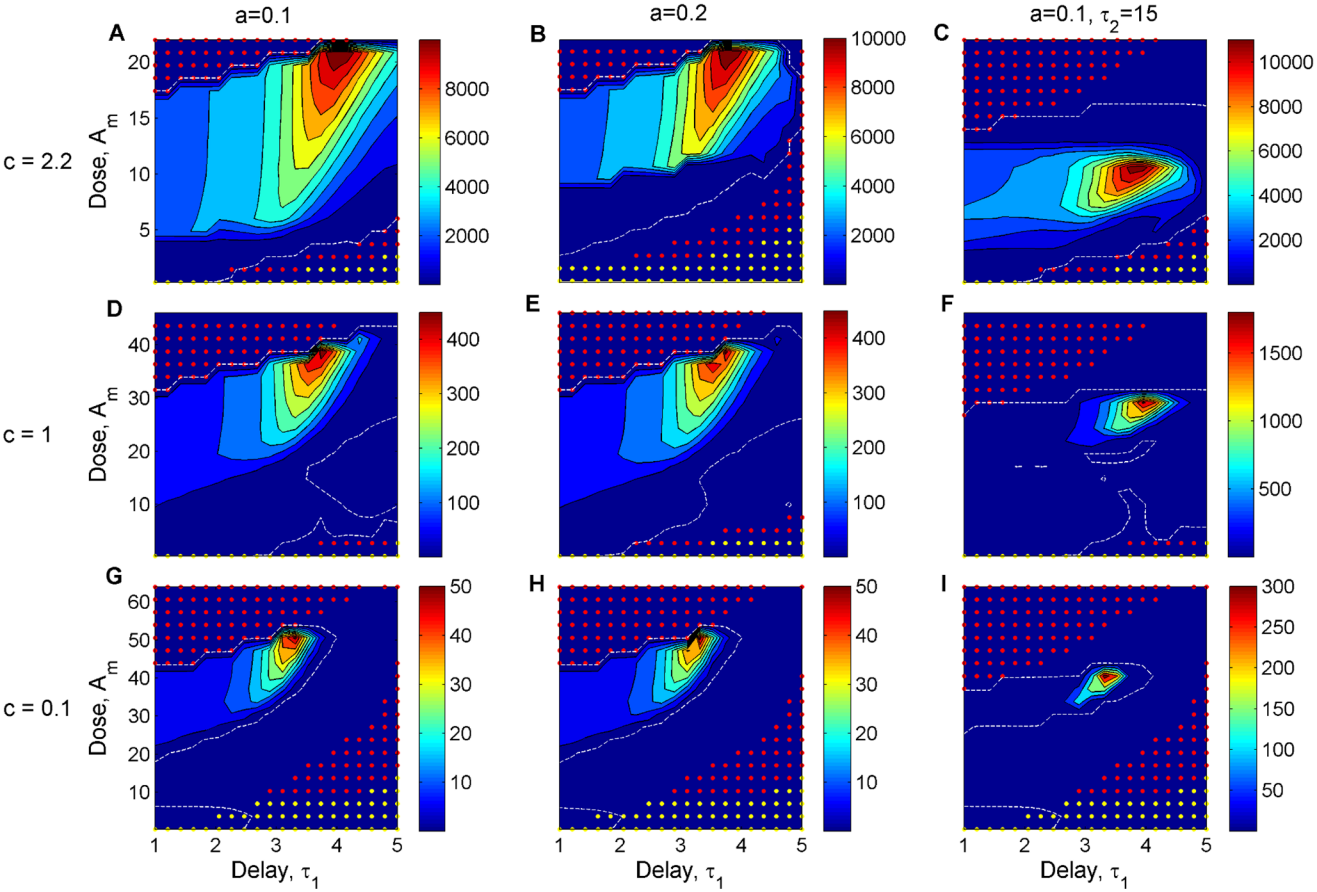
Resistance selection over treated infections, for different combinations of dose *A*_*m*_ and delay *τ*_1_ in the classical regime. The simulated values of *A*_*m*_ correspond to 30 doses in the range $\left[0.1A_{m}^{*},2A_{m}^{**}\right]$. We plot the proportional change in the resistance burden over treated infection *R*_*tot*_, relative to the same measure in untreated infection, as a function of antibiotic dose and timing of treatment onset for a range of infection scenarios. In the subpanels, the benefit and cost of resistance, and treatment duration vary as: A) *a* = 0.1, *c* = 2.2, *τ*_2_ = 7; B) *a* = 0.2, *c* = 2.2, *τ*_2_ = 7; C) *a* = 0.1, *c* = 2.2, *τ*_2_ = 15; D) *a* = 0.1, *c* = 1, *τ*_2_ = 7; E) *a* = 0.2, *c* = 1, *τ*_2_ = 7; F) *a* = 0.1, *c* = 1, *τ*_2_ = 15; G) *a* = 0.1, *c* = 0.1, *τ*_2_ = 7; H) *a* = 0.2, *c* = 0.1, *τ*_2_ = 7; I) *a* = 0.1, *c* = 0.1, *τ*_2_ = 15. The resistance selection window is defined by the dashed white line (contour line corresponding to a proportional change of 1). Superimposed are treatment combinations that maintain infection duration within a factor of 1.1 relative to no treatment (yellow dots), and those that, in addition, satisfy a reduction in immunopathology by at least 2-logs order of magnitude (red dots). All parameters as in Table 1, unless otherwise stated. The growth rate of the resistant bacteria *B*_*r*_ is varied as *r*_1_ = *r*_0_ − *c* (different rows). Moderate dose-delay combinations, applied below the resistance selection window at each infection, can be effective in clearing the pathogen, in synergy with host immune responses. The aggressive high doses instead are effective without relying on the contribution by the immune system.

When mapping each infection profile to a quantitative assessment of infection summary measures (see Methods), we notice more clearly how treatment dose and delay mediate selection for resistance, infection duration and host immunopathology, as shown in S3 Fig. In Fig 5 instead, we illustrate primarily one dimension of treatment success: resistance selection. Considering several treatment landscapes, we observe that for intermediate doses, there is a selection window for resistant bacteria, namely mutant selection window (MSW), that shifts when treatment onset is delayed. This selection window includes a much narrower range of doses when the susceptibility *a* of the resistant strain increases, or when treatment duration increases from 7 to 15 days. With diminishing cost of resistance, the resistance selection window moves towards higher doses, as expected.

In particular, Fig 5 shows the relative selection of resistance in different infections under treatment, varying the cost and benefit of resistance, and treatment dose, timing and duration. The doses are chosen in each case to represent the relevant relative range interpolating $\left[0.1A_{m}^{*},2A_{m}^{**}\right]$, thus comprising doses below, within and above the critical inhibitory doses for *B*_*s*_ and *B*_*r*_ respectively. The resistance selection window, depicting dose-delay combinations in which *R*_*tot*_ is higher than the resistance burden of an untreated infection is given by the white dashed contour line, and shown separately for clarity also in S4 Fig.

In such landscapes, one can seek optimal treatment by imposing that certain targets be met in terms of infection features, taking as reference the corresponding untreated infection. For illustration, we first set a target for treatment to successfully clear infection: quantitatively, to keep infection duration within a factor of 1.1 relative to its value in the no-treatment case. The resulting dose-delay combinations are depicted with yellow dots. Then, we set in addition a second target: for treatment to lower immunopathology *H*_*tot*_ by at least 2-log orders of magnitude. Those combinations of dose and delay that satisfy both these criteria are depicted by red dots.

Among these candidates, we can notice that there are broadly two ways of reaching infection clearance and reducing immunopathology with classical treatment: either with supercritical doses, above the resistance selection window, or with subcritical doses, below the resistance selection window. In the first case, there is little or no involvement of host immunity, whereas in the second case, there is synergistic immune response contribution to pathogen clearance. In the latter moderate treatments, as treatment is delayed, a greater level of host immunity is expected at onset, thus there is less chance for immunity to fall to subcritical levels during treatment. For this reason, with later onsets, we observe that higher doses begin to satisfy our optimality criteria. Although they are not needed for clearance, slightly higher doses, in this moderate range, remove the bacterial killing burden from the immune system, yielding lower pathology. Even when the cost of pre-existent resistance is lower (Fig 5D and 5G), moderate doses, below the corresponding $A_{m}^{**}$, applied at moderate delays post-infection, can be effective to rapidly remove the pathogen, limiting the ascent of the resistant sub-population.

Regarding the impact of longer classical treatment, 15 days, as opposed to 7 days, we find that in the moderate dose range (below the selection window) prolonging the therapy does not add new optimal dose-delay combinations (Fig 5), and no significant gains in infection outcomes such as resistance selection or time to clearance. In contrast, in the aggressive dose range (above the MSW), longer duration of treatment increases the effectiveness of relatively lower doses: expanding the range of small dose-delay combinations that can be used to clear infection (shown in S5 Fig), as expected from our earlier analysis in Eq 19.

**Fig 6:**
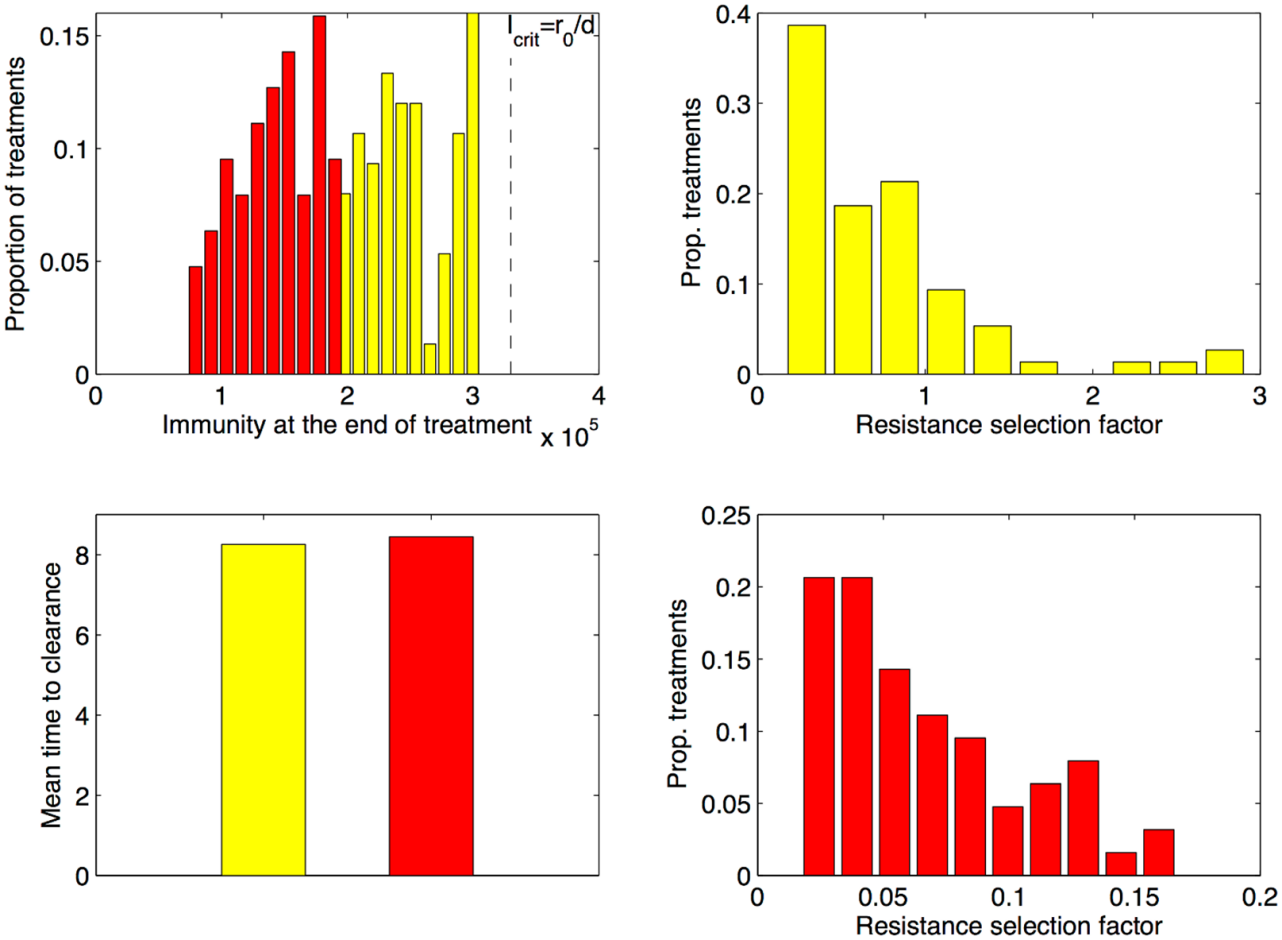
Details from optimal moderate treatments in the classical case, for high cost of resistance. We compare infection outcomes obtained from selected dose-delay combinations (A_*m*_, *τ*_1_) below MSW in scenario G of Fig 5 where the cost of resistance is *c* = 0.1. The treatment combinations satisfying only the duration criterion (yellow) display higher immunity levels at the end of infection than treatment combinations satisfying both the low duration and low immunopathology criteria (red). The resistance selection factor (relative to no treatment) is lower in scenarios of low immunopathology. However, as shown in the top-right panel, in about 75% of the other moderate scenarios (yellow), the resistance selection factor is still below 1, evidently marking an overall reduction compared to no- treatment.

Zooming further into optimal treatments, (e.g. the low cost resistance scenario of Fig 5G), we find that dose-delay combinations satisfying only the duration criterion (yellow) yield higher immunization levels for the host at the end of infection, than treatment combinations satisfying both the low duration and low immunopathology criteria (red), shown in Fig 6. Resistance selection, compared to untreated infection, is also lower in scenarios of low immunopathology. However, in a majority of the other moderate scenarios, shown in the top-right panel in Fig 6, the resistance selection factor is still below 1, evidently displaying an improvement compared to no-treatment.

Unsurprisingly, when immunity does not wane (*h* = 0), clearance of infection by classical treatment becomes easier, (S6 Fig) and the range of effective moderate doses below the MSW increases slightly. In this case, the MSW peak is lower than that in the *h* > 0 case, illustrating reduced potential for pathogen growth within-host, and it is centred at earlier delays, due to a more robust immune response experiencing only minor interference by treatment.

Taken together, these results confirm the effectiveness of aggressive therapy, but also uncover the effectiveness of moderate antibiotic doses, in combination with appropriate timing of treatment, for achieving synergistic infection clearance involving host immunity.

### Adaptive treatment and the role of the symptom threshold

After considering classical treatment, with fixed onset, duration and dose, we also explore infection dynamics under an adaptive treatment regime (Fig 1C), where drug uptake is related to pathogen density (*B*(*t*) ≥ Ω). Here we vary the dose *A*_*m*_ and the symptom threshold Ω, i.e. the total pathogen load above which the host takes the antimicrobial drug.

**Fig 7:**
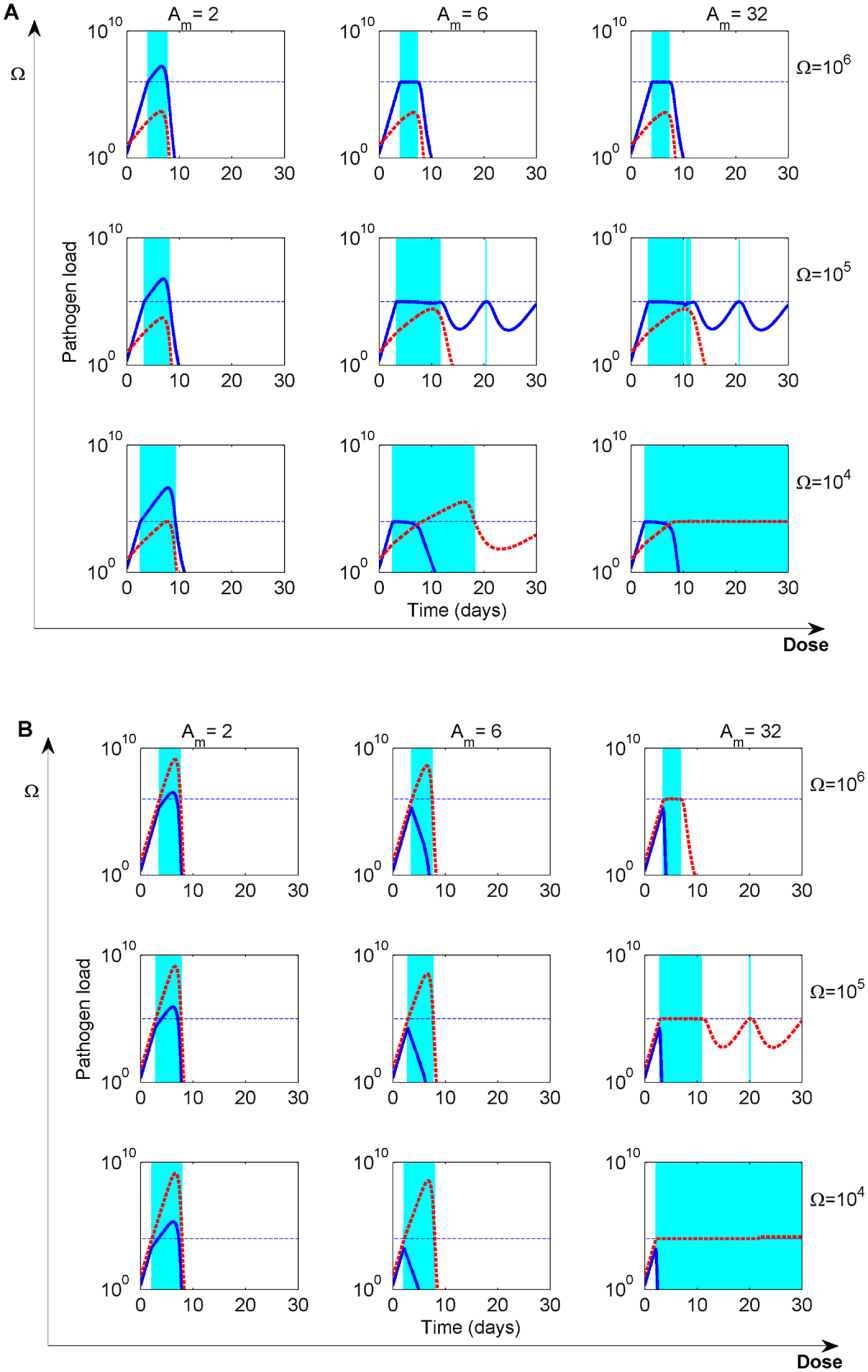
Adaptive treatment dynamics depends on dose *A*_*m*_ and the symptom threshold Ω. **A)** High cost of resistance (*c* = 2.2) where $\left[A_{m}^{*},A_{m}^{**}]=[3.3,11\right]$. **B)** Low cost of resistance (*c* = 0.1) where $\left[A_{m}^{*},A_{m}^{**}]=[3.3,32\right]$. All other parameters as in Table 1 (*r*_0_ = 3.3, *k* = 10^5^). Drug-sensitive pathogen dynamics are plotted in blue solid lines, and drug-resistant pathogen dynamics are given by the red dashed lines. In adaptive treatment, doses below $A_{m}^{*}$ allow bacterial growth during treatment thus co-stimulation of immunity. Higher doses are scaled according to pathogen density *B*(*t*) ≥ Ω, which maintains the total infection load at Ω, until sufficient host immunity has been mounted. Doses above $A_{m}^{**}$ can be scaled down if necessary (A top right panel), or used in full (B top panel) guaranteeing the no-growth condition. Under *σ* = 2 as in Table 1, at too low symptom thresholds (Ω < *k*), the treatment starts too early, and immunity does not reach the critical level. When coupled with high dosage of treatment, this results in chronic infection being maintained indefinitely, unless other killing mechanisms or non-specific immune defenses are present at such low bacterial densities and can assist in clearance.

Our simulations convey that how the symptom threshold Ω compares with the host immunity threshold *k* impacts strongly duration of treatment and the ensuing dynamics (Fig 7). Recall that *k* corresponds to the pathogen density required for half-maximal immune stimulation. We observe that for Ω ≤ *k*, the drug starts to act too early, namely before or just when host immunity has been triggered to grow at its half-maximal rate, and adaptive treatment does not always clear an infection. In particular, if high doses are applied too early, an adaptive regime leads to chronic maintenance of either resistant or sensitive bacteria, in relapsing mode, peaking at Ω for indefinite time, an effect due to suboptimal immune activation and direct coupling of treatment to pathogen load. If treatment allows for minimal pathogen growth, thus only at low doses, adaptive treatment can yield pathogen clearance.

**Fig 8:**
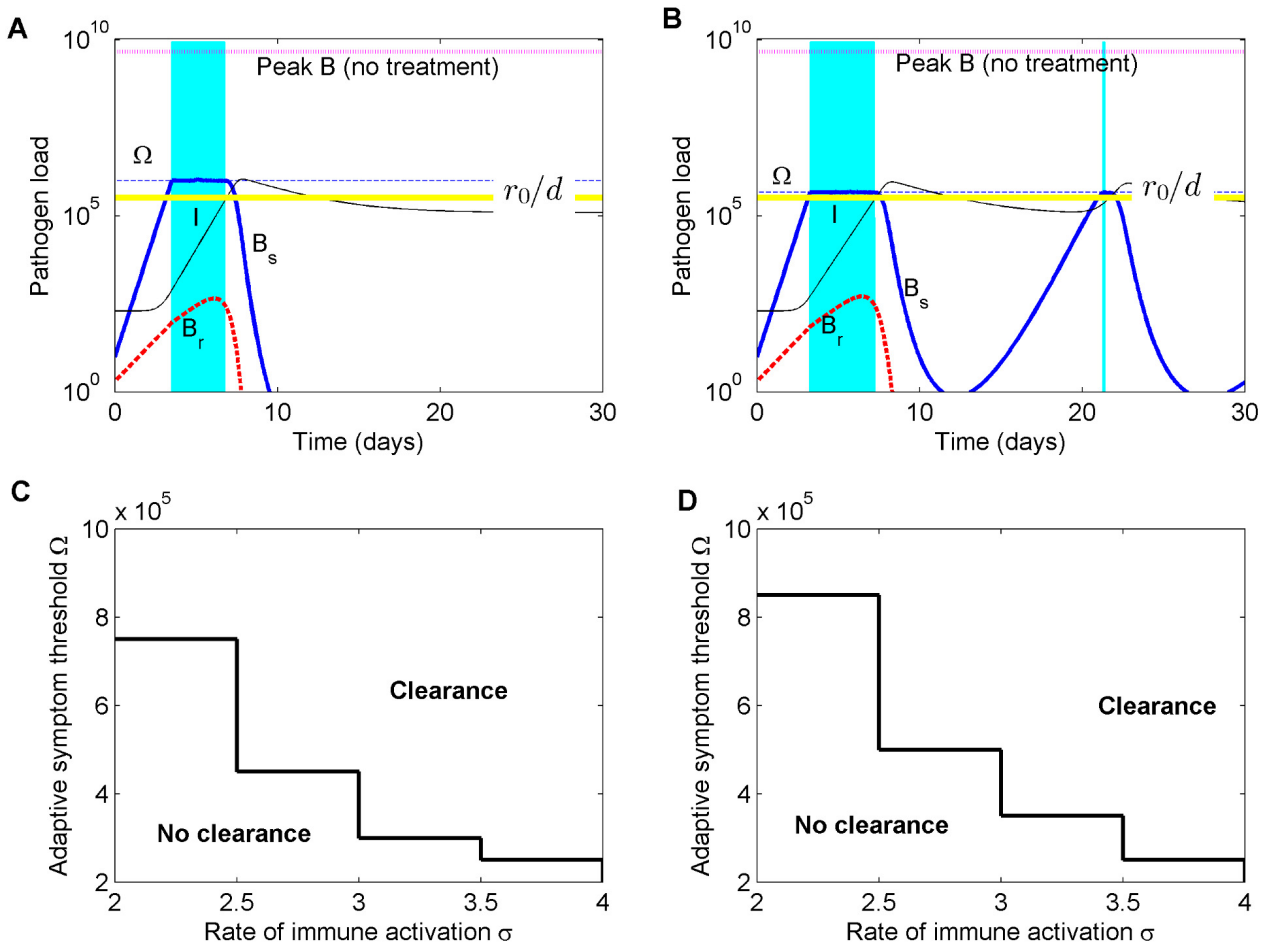
Illustration of adaptive treatment above the immunity threshold (Ω > *k*). **A)** Clearance dynamics when Ω is sufficiently high (Ω = 10*k*), i.e. satisfies Eq 22. **B)** No clearance dynamics, when Ω is too close to *k* (Ω = 5.6*k*), and adaptive treatment induces oscillatory dynamics. **C)** High rate of effector cell conversion into memory *f* = 0.1. **D)** Lower rate of conversion into memory *f* = 0.05. When persistent immunity accumulates faster, the range of symptom thresholds where adaptive treatment works, approaches the host immunity threshold, enabling treatment success at lower pathogen densities. Parameters as in Table 1, with *k* = 10^5^.

On the other hand, when the symptom threshold exceeds the immunity threshold (Ω > *k*), effective clearance of infections is more likely to occur (Fig 8A). In these cases the immune system and the drug possibly act in synergy, for as long as needed to initiate and complete bacterial clearance. During such an adaptive treatment, sensitive bacteria can continue to grow slightly above Ω if doses are low, or remain fixed at Ω if doses are higher, but selection of the resistant sub-population is minimal and independent of the dose.

Intuitively, for the success of the adaptive strategy, the involvement of the immune system is a pre-requisite. However, how high exactly the ratio Ω/*k* needs to be, in order to guarantee clearance, must depend on the magnitudes of other immunity parameters, such as *σ* and *h*. For example, the higher the rate of immune stimulation, *σ*, or rate of memory formation, *h* *f* the closer to *k* the adaptive symptom threshold can be (i.e. the earlier treatment can begin). We make this precise by the following arguments.

Suppose that when treatment begins, thus when *B*(*t*) = Ω, most of the bacterial population consists of drug-sensitive bacteria (i.e. when *r*_0_ > *r*_1_). Then the dose required to interrupt bacterial growth is roughly $A_{m}=\frac{r_{0}-dI(t_{\Omega })}{\delta_{0}}$. Conversely, if *r*_0_ ≈ *r*_1_, and the fitness cost of resistance is low, this no-growth dose is determined by the resistant sub-population $A_{m}=\frac{r_{1}-dI(t_{\Omega })}{a\delta_{0}}$.

By instantaneously modulating rate of drug administration *η*(*t*), adaptive treatment manages to effectively scale higher antibiotic doses to these minimal inhibitory values, thereby maintaining pathogen load at Ω. Optimal adaptive treatment, during drug-administration, keeps *B*(*t*) at the symptom threshold Ω, which is sufficient to provide constant immune stimulation. Under this scenario, using Eqs 4 and 5, the immune response dynamics during treatment can be approximated as:

$$\begin{array}{ccc} I\left(t\right) & \approx & E\left(t\right)+M\left(t\right)\\ & = & E\left(t\right)+hf\left(1-\frac{\Omega }{\Omega +k}\right)\overset{t}{\underset{t_{\Omega }}{\int }}E\left(s\right)ds\\ & = & I\left(t_{\Omega }\right)e^{Zt}+hf\left(1-\frac{\Omega }{\Omega +k}\right)\frac{I\left(t_{\Omega }\right)}{Z}\left(e^{Zt}-1\right), \end{array}$$ (20)

where, for simplicity of notation

$$\begin{array}{c} Z\equiv \left(\sigma +h\right)\frac{\Omega }{\Omega +k}-h \end{array}$$

represents the net exponential rate of change, and *I*(*t*_Ω_) is the immunity at treatment onset, i.e. when *B*(*t*) reaches Ω. This level of immunity at treatment onset can be computed from the approximation in Eq 13, replacing *B* by Ω and solving for *I*. In the derivation of Eq 20, the contribution of *N* cells is assumed negligible, and total immune response is given by effector and memory cells only. The condition for the exponential rate of change to be positive (*Z* > 0) is satisfied if: Ω/*k* > *h*/*σ*. It becomes evident that the rate of growth of the host immune response during treatment depends not only on how Ω compares with *k*, but also on the lymphocyte recruitment rate *σ*, rate of decay *h*, and proportion differentiating into persistent memory cells *f*. Clearly, the same rate of immune buildup may be achieved by the balanced effects of either higher Ω/*k* or higher *σ* (see S7 Fig).

Enabling the immune system to catch up during treatment, the adaptive therapy will then last until the critical level of immunity, *I*_*crit*_, has been reached. Using Eq 20, the expected duration of successful adaptive treatment can be calculated as:

$$\begin{array}{c} {\text{Dur}}_{adaptive}=\frac{1}{Z}\log\left[\frac{\frac{I_{crit}}{I(t_{\Omega })}Z+hf\left(1-\frac{\Omega }{\Omega +k}\right)}{Z+hf(1-\frac{\Omega }{\Omega +k})}\right], \end{array}$$ (21)

after which the immune system of the host should be able to finish the ‘job’ of pathogen clearance. From this equation, matching very well with our simulations (S8 Fig), we observe that a stronger immune response guarantees shorter treatment duration, during which the drug is taken only while necessary, to facilitate subsequent action by the immune system.

At the end of such an adaptive treatment, the host immune responses have reached the critical level *I*_*crit*_, required for initiating pathogen decline. Although this is the first necessary step for clearance, it is not sufficient. While pathogen load starts to decline from level Ω, residual immune stimulation continues initially post-treatment, as long as total pathogen load satisfies $B>\frac{kh(1-f)E}{\sigma (N+E)}\approx \frac{kh(1-f)}{\sigma }$, following from Eqs 3–5. But as *B* drops to low numbers, the immune response starts to decline as well. If the time it takes for this declining immunity to reach *I*_*crit*_ again, exceeds the time it takes for the declining pathogen load to hit the extinction threshold, then clearance occurs after adaptive treatment (Fig 8A). On the contrary, infection clearance does not occur if immune responses fall to sub-critical levels before pathogen extinction (Fig 8B). In that case, an oscillatory dynamics between pathogen—occasional treatment—and immunity emerges, and infection continues. Mathematically, by analyzing the *‘contraction’ phase* of host immunity, these clearance and no-clearance regimes can be approximately distinguished via the following criterion (see S1 Text, part II):

$$\begin{array}{c} \frac{\log\left(\frac{B}{\Omega }\right)}{\log\left(\frac{B+k}{\Omega +k}\right)}=\frac{\sigma }{h(1-f)}+1\;\text{and}\;\left\{\begin{array}{cc} B\leq B_{ext}\to & \;\text{clearance}\\ B>B_{ext}\to & \;\text{relapse,} \end{array}\right. \end{array}$$ (22)

which applies to the pathogen load when host immunity hits *I*_*crit*_ during its contraction phase. If the solution to the above equation is below the extinction density *B*_*ext*_, then adaptive treatment administered at symptom threshold Ω will be effective, otherwise oscillatory dynamics will be induced by treatment instead of clearance. The above conditions specify the parameter space where adaptive treatment can provide a sustainable solution for resistant infections under minimal drug pressure, highlighting the important role of the immunity threshold *k*, as well as other immunity parameters. Thus, not all symptom thresholds Ω above *k* are effective: only those, which in combination with the rest of immune indicators, and the pathogen extinction threshold, satisfy the necessary and sufficient criteria for clearance, as illustrated in Fig 8C and 8D.

Notice that the distinction between clearance and relapse after adaptive treatment, obviously, only applies if there is the possibility of a contraction of the immune response in the time scale of interest, i.e. if *h* > 0. In case immunity does not wane (*h* = 0), clearance always follows after adaptive therapy, with ever-increasing immunity *I* > *I*_*crit*_, and Eqs 20 and 21 still apply. What may vary is the speed of such clearance, ultimately regulated by the pathogen killing efficiency of immune cells, *d*.

### Classical vs. adaptive regime?

**Fig 9:**
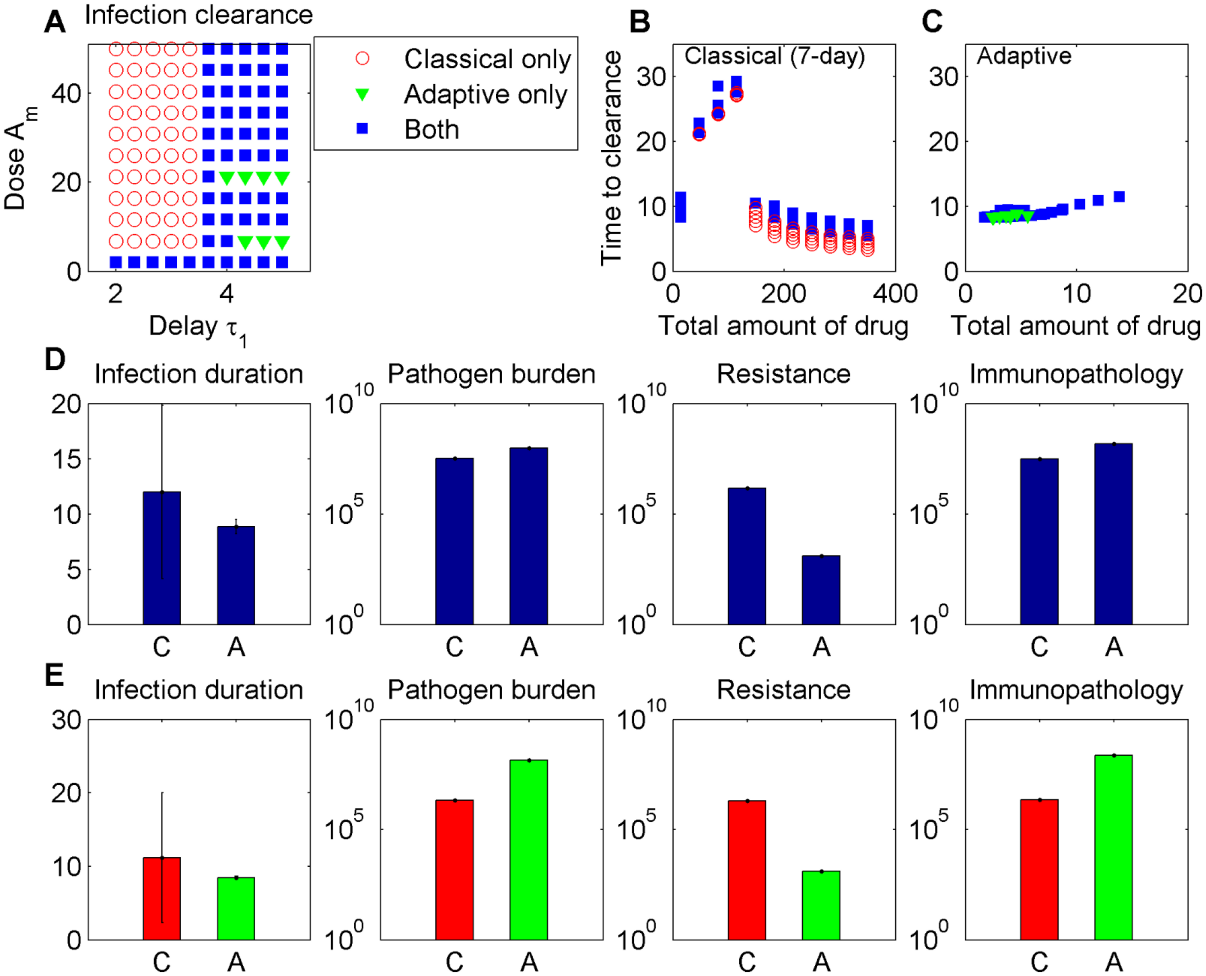
Comparison of classical and adaptive regimes, for variable dose and treatment delay. Different treatment scenarios are simulated up to *T* = 30 days post-infection. Classical treatment assumes a fixed duration of 7 days, while in the adaptive regime, drug uptake is related to bacterial density above the threshold Ω = *B*(*τ*_1_). Parameters as in Table 1. **A)** Clearance of infection by 30 days can be obtained: via classical treatment only (red), via adaptive treatment only (green) or using either regime (blue). **B)-C)** The amount of drug deployed in each treatment and the associated time to clearance. Longer time to clearance in the classical regime at low doses corresponds to relapsing infection after treatment cessation, and delayed clearance by host immunity. **D)** Treatment outcomes in those cases where both regimes, classical/C, and adaptive/A, can yield infection clearance (mean ± sd), with: ${\bar{B}}_{tot}^{C}=3.2\times 10^{7}$, ${\bar{B}}_{tot}^{A}=9.6\times 10^{7}$; ${\bar{R}}_{tot}^{C}=1.4\times 10^{6}$, ${\bar{R}}_{tot}^{A}=1.3\times 10^{3}$; ${\bar{H}}_{tot}^{C}=3\times 10^{7}$, ${\bar{H}}_{tot}^{A}=1.5\times 10^{8}$. **E)** Treatment outcomes in those cases where clearance can be achieved exclusively via one or the other regime (mean ± sd), with: ${\bar{B}}_{tot}^{C}=2.1\times 10^{6}$, ${\bar{B}}_{tot}^{A}=1.4\times 10^{8}$; ${\bar{R}}_{tot}^{C}=2.0\times 10^{6}$, ${\bar{R}}_{tot}^{A}=1.3\times 10^{3}$; ${\bar{H}}_{tot}^{C}=2.2\times 10^{6}$, ${\bar{H}}_{tot}^{A}=2.3\times 10^{8}$.

To jointly evaluate both protocols on the same infection, we assume *τ*_1_ (classical treatment) equals the time it takes the total bacterial population to reach the symptom threshold Ω (adaptive treatment) in the absence of the antibiotic, so we ensure equivalent treatment onsets. We consider variable symptom threshold Ω = *B*(*τ*_1_), and variable dose *A*_*m*_. Assuming a fixed duration of 7 days for classical treatment, we compare infection outcomes between the two regimes (Fig 9), for simulated dynamics up to 30 days.

Keeping all other immunity parameters fixed, when the symptom threshold is lower than or equal to the immunity threshold, it is classical treatment with high doses that is more likely to clear infection, by 10 days on average, as shown in Fig 9A (red circles). At such low bacterial densities adaptive treatment generally fails, and induces oscillatory pathogen dynamics, unless the doses are sub-inhibitory, such that bacterial growth continues during treatment, stimulating immunity and eventual clearance.

As the delay of treatment onset increases, implying higher symptom thresholds, clearance of the same infection can be obtained by both classical and adaptive regimes (Fig 9A, blue squares). However, while in the classical regime, the clearance doses are generally very high, the effective doses in adaptive therapy are much lower (Fig 9B). Contrary to the fixed protocol of the classical regime, in adaptive treatment, duration and drug uptake are modulated by pathogen load. As immune response grows rapidly during adaptive treatment at symptom thresholds above *k*, decreasing the growth potential of bacteria within host, the rate of uptake of the drug progressively goes down. This effectively reduces the dosage of therapy to the minimum necessary, thereby limiting resistance selection, and minimizing gradually any interference with immunity.

For our illustrative parameter values (Table 1), as shown in Fig 9C, adaptive treatment achieves infection clearance by day 8 post-infection, averaged over all dose-delay combinations, while classical treatment clears infection later, in 12 days on average. This longer time to clearance results from those cases when low-dose classical therapy disrupts temporarily the natural course of host immune defenses, leading to bacterial relapse post-treatment and delayed immune control.

The differences in immunopathology and total bacterial burden from our simulations favour classical treatment by a slight amount, but when considering total resistance burden, the adaptive treatment is remarkably superior, yielding lower values by at least two orders of magnitude, averaged across all dose-delay combinations that we tested. The analogous comparison between the two protocols, for a lower cost of resistance, is shown in S9 Fig, where the greater immune activation obtained from resistant bacteria growing slightly at sub-inhibitory doses, increases the success potential for adaptive treatment, but at the cost of higher pathology. We also find treatment scenarios where infection may be prolonged indefinitely, both within classical and adaptive regimes, a failure feature attributed previously only to adaptive scenarios [31].

As the distance of the symptom threshold from the immunity threshold (Ω/*k*) increases, treatment is initiated at higher and higher pathogen loads, where the preponderate role in bacterial killing can be more readily fulfilled by the immune system. At such high pathogen numbers, classical treatment, by maintaining drug pressure over longer periods of time, relieves to a larger extent the burden on host defenses. Thus, a classical regime is bound to yield lower immunopathology, because it replaces the role of the host’s immune system. Adaptive treatment, instead, does precisely the opposite: it exploits the contribution of the immune system, and while doing so, is able to achieve the same reduction in pathogen load and infection duration. On one hand, this might entail the cost of more pathology, but also brings about the potential benefit of host immunization.

## Discussion

An essential question in resistance management is how can evolutionary approaches be applied to identify therapeutic regimes that can best counteract the rise of antibiotic resistant pathogens [14, 15, 60]. Optimal treatment protocols should seek to restore patient health, while minimizing immunopathology, limit the pathogen load and resistance over infection, and prevent transmission. In this paper, we have used a mathematical model to examine conditions of antibiotic treatment that optimize outcomes of infection with drug-resistant infectious organisms. Although the role of immunity and treatment timing has been evoked in recent work, in this paper we have provided a deeper analysis of these two important aspects of treatment, in particular interpolating between classical and adaptive regimes.

Motivated by recent studies on the topic [14, 30, 31], we have explored in detail the fine interplay between antibiotic dosage, treatment onset and duration, and host immunity. The interaction between antibiotics and the immune response can be antagonistic when antibiotic-mediated pathogen killing decreases the intensity of the immune response activated for infection clearance. However, we find that appropriate timing, moderate dosage and duration of treatment can transform this interaction into a synergistic one, where both antibiotic and host immunity act together.

Antibiotic treatment with fixed dose and duration is the dominant conceptual framework in antimicrobial therapy and clinical settings. Recently it has been questioned whether the classical recommendation of treating infections as aggressively as possible, with high antimicrobial doses and long duration, is the most appropriate therapeutic strategy in the presence of drug-resistance [14, 16]. Due to the growing resistance crisis, clinical practice is also opening up to treatment regimes that offer either a more dynamic alternative to fixed prescription, or explore delaying treatment onsets [61–64]. In the present study, analysis of our model and changes in several key parameters, allows us to go a step further in this discussion. We observe that the reality of within-host infection dynamics is highly non-linear, with many intervening pressures among which host immunity, that act in conjunction to produce diverse outcomes. We have dissected the effects of classical therapy, being it aggressive or moderate. Further, we have identified the mechanistic links between moderate treatment and an adaptive regime, where drug uptake closely follows infection progression via symptom signalling of pathogen dynamics.

We have found that whether in a classical or adaptive regime, timing of antimicrobial therapy is very important for treatment success. Treatment timing (delay) effects are twofold: on one hand related to pathogen load, and on the other hand, to the level of immunity expected upon onset. While increasing pathogen load requires higher doses to achieve clearance by a given time, increasing immunity over the course of an infection acts to facilitate clearance with smaller doses. These two opposing forces can be reconciled through intermediate treatment timing, corresponding to pathogen loads just above the immunity threshold (the half-saturation constant for antigen stimulation of immunity). Undoubtedly, this optimal timing is subject to other immune characteristics and pathogen factors, and we have analyzed in detail some of these here, including the rate of immune activation, decay and differentiation into memory. While different pathogen systems may exhibit different forms of interaction with immunity, requiring adaptation of model structure, similar critical drivers of treatment success, as the ones analyzed here, may emerge.

Besides the *‘hit hard and fast’* protocol, we find that classical treatments of moderate doses and moderate duration, when applied at the right time, can promote a synergy between the host immune system and antibiotic treatment. The definition of ‘moderate’ depends on the critical dose range $\left[A_{m}^{*},A_{m}^{**}\right]$, that varies with fitness cost and benefit of resistance, and with absolute pathogen growth rate within host. Adaptive treatment, on the other hand, calibrates moderation in an autonomous manner, by coupling drug administration to pathogen dynamics, and its typical effectiveness in leading to infection clearance depends on how the symptom threshold compares to the host immunity threshold and other kinetic parameters. A safe choice in adaptive regimens seems to be deployment of the higher $A_{m}^{**}$ dose as default (minimal inhibitory dose for the resistant sub-population), which will be naturally scaled down to $A_{m}^{*}$ in the best case, or will be taken in full if necessary in the worst case. The duration and success of adaptive therapy is then naturally determined by the strength and speed of host immunity, suggesting that it may not be a feasible solution for immunocompromised hosts.

When comparing the performance of the classical and adaptive regime in treating the same infection, we found again that the timing in relation to the immunity threshold *k*, was important, although the critical value is inevitably modulated by other host immunity parameters. Below the immunity threshold, typically only the classical regime could clear an infection by 30 days, or only adaptive regimes with sub-critical doses, where pathogen levels stimulate sufficient immunity. Generally, above the immunity threshold, adaptive and classical treatment can both be effective in clearing infections, but in different ways, and using different amounts of antibiotic pressure. While classical treatment deploys higher amounts of drug, and is generally superior in terms of yielding lower host immunopathology, adaptive treatment, by relying on minimal drug pressure, minimizes selection of pre-existing resistance and promotes immunization.

The importance of immunization depends on the likelihood or re-exposure to the same pathogen. While the level of immunity provided by moderate treatments may yet be sub-optimal to completely clear a secondary infection (see S10 Fig), it may be sufficient to constrain growth, trigger a rapid secondary response, or contribute to clearance alongside innate immunity or other control mechanisms. The caveat for the adaptive strategy remains ensuring perfect translation and immediate response delivery to the symptom signal, and here more research is needed. In reality, one is usually constrained by how much immunopathology a given host can tolerate, how toxic the drug is, and also what upper bounds one should target for overall resistance and infection duration. Hence, there may be cases where the dose needed to effectively eliminate all pathogens might also not be an available option.

### Links with empirical data

By exploring in detail the treatment parameter space, across classical and adaptive regimes, we uncover the cases where a more prudent use of antibiotics, with lower doses over less time, could be favoured, as a more effective way to reduce the rate of ascent of resistance. Our results thus extend the perspectives of Read et al. [14], highlighting the novel important role of treatment timing, as advocated recently by other modeling studies [23, 33], and bring forward new testable hypotheses.

Our model behaviour illustrates four broad infection outcomes: acute infections dominated by either drug-sensitive (i) or resistant bacteria (ii), and relapsing infections dominated by drug-sensitive (iii) or resistant bacteria (iv), which can reach higher or lower peaks than pre-treatment levels. Relapsing resistant infection, has been observed experimentally in malaria parasites *in vivo*[16] and in bacterial infections of humans, e.g. urinary tract recurrent infections within 2 weeks of treatment [65], infections with *C. difficile*[66], in clinical treatment of *Salmonella* bacteraemia [67] and in treatment failure of endocarditis [68], where relapse was associated to heterogeneous resistance of *Staphylococcus aureus* pre-treatment. Treatment failures leading to relapses often occur because of a mismatch between first line antimicrobial therapy and the unknown antibiotic susceptibility of the disease-causing agent. On a broader level, infection relapses post-treatment may have parallels with the trade-off between clearance by intervention and immunization, observed also at the epidemiological level, as it has been argued for example in schistosomiasis control [69], where massive drug administration, reducing antigen exposure, could result in rapid decline of protective immunity in the population and infection rebound if treatment ceased.

To successfully treat resistant infection in hosts with an intact immune system, our model suggests that doses can be moderate, especially when the treatment (symptom) threshold exceeds the immunity threshold. This results in an optimal delay [33], after which the critical pathogen density, triggering adaptive immunity, has been surpassed, and the subsequent overshoot in pathogen-growth is minimized through treatment. This is similar to the principles advocated by Tanaka et al. [23] for treatment timing near the epidemic peak at the population level. Our results thus highlight the importance of appropriate timing of moderate therapy and synergistic action of host immunity whenever possible. These theoretical analyses corroborate evidence from experimental and clinical studies of infection [40, 70, 71], and call attention on the empirical values of the host immunity threshold [36], and other variable immune response determinants [72] as critical modulators of treatment success.

### The role of mutational input to the system

In our model, we neglected the process of *de novo* mutation that can lead to resistance to the current drug. Exploiting the analogy with the Luria-Delbruck process [73], it is known that the timing at which the first resistant clade arises impacts strongly on the subsequent prevalence of resistance in a growing bacterial population. If the first resistant strain arises early over infection, a large resistance clade ensues within host. In contrast, if the first resistant strain arises later, the resulting clade is expected to be smaller. These effects are amplified in the presence of immune control. A similar scenario at the population level has been explored by Tanaka et al. [23]. In a way, our model considers the worst extreme of this timing spectrum for the host, by assuming that resistance is already pre-existent from the start of infection. Assuming some fitness cost of resistance and a slight advantage in initial numbers of the drug-sensitive bacteria, postponing slightly antibiotic treatment until host immunity is triggered, gives the sensitive sub-population a sufficient head start, limiting the ascent of the resistant competitors.

It is possible that during infection, *de novo* resistance emergence may occur, at any point of the dynamics. Moreover, the probability of *de-novo* emergence of resistance through a mutation rate per unit of time should apply to each parental strain: the sensitive and resistant bacteria. In fact, it has been shown that already-resistant strains may have higher mutation rates to develop new resistances [74]. However, to describe the subsequent dynamics of these newly emergent strains within host, one would need to make further assumptions about what the cost and fitness benefits of such new mutations may be with regards to the present infection. In this process, one may choose to explicitly simulate emergence, or could track, for example, the overall probability of new (generic) resistance emergence over the entire infection period, $P_{emergence}=1-e^{-\theta \overset{D}{\underset{0}{\int }}B(t)dt}$, with *θ* denoting mutation rate per unit of time, and examine criteria to restrict this quantity. Following [75], a critical threshold for this probability could be the value 1 − *e*^−1^, above which emergence would be expected almost certainly in a semi-stochastic setting. To constrain such probability of emergence, basically implies a restriction on the total bacterial burden over infection, in relation to the particular value of the mutation rate, and this could be readily investigated with the current framework.

In the future, it will be essential to address the process of mutational input to the system in more detail, and analyze the different phenotypes that may emerge, their mutual correlation, and their consequences for infection dynamics. These include drug resistance to the current and other drugs [74], with the worst scenario being an increase in non-susceptibility (1 − *a* parameter in our model), persister phenotypes [76], compensatory mutations reducing fitness cost [34], virulence, and interaction with the host immune system. It is likely that some of our findings may not hold in all scenarios, and this remains an open avenue, to be explored on a case-by-case basis. In specific bacterial systems displaying drug resistance, empirically derived distributions of mutation effects [77] can eventually be used to inform realistic model extensions. Another line of exploration remains the process of horizontal gene transfer, where the rates of resistance exchange through mobile elements have been shown to be orders of magnitude higher than those of point mutation [78].

### Future prospects

In the interest of generality and clarity, several simplifications were necessary to obtain analytical insight. Focusing on the principal fitness differences between resistant and sensitive bacteria, we did not further consider separate in-host compartments for bacterial growth as in [31]. We also focused on exponentially growing pathogen in the absence of treatment, although previous studies have accounted for logistic growth imposed by resource limitation [24, 30, 31]. Logistic growth changes the dynamics of untreated infections in a major qualitative manner, allowing for acute infections, as well as persistent colonization states, typically observed with enteric bacteria [79]. Among other issues, this qualitative change would require a rethinking of the optimality criteria for antibiotic treatment, and of the interplay with host defenses. We believe an understanding of how logistic growth parameters may interact with the host immunity threshold and treatment parameters deserves a deeper study of its own, where systematic analysis of different scenarios can reveal new results.

Using a deterministic approach, in line with existing studies on the topic [29, 30], we also did not explicitly model demographic stochasticity of bacteria within host, beyond the assumption of an extinction threshold. We decided to focus on understanding the mechanisms of infection dynamics and the selective processes operating during treatment. Naturally, for greater realism and applicability of the model in practical settings, all factors contributing to stochasticity and bacterial growth in different organs and host compartments must be accounted for in the future (see [80] for a data-driven example on *Salmonella* infection, and [81] for a recent computational tool developed for TB treatment). As previous models have shown, drug concentration heterogeneity in different body compartments may yet be another factor impacting drug resistance evolution within host [82].

Notice that we do not deal with secondary infections in this paper. Immune kinetics over consecutive encounters with the same pathogen is complex, and the precise relationship between memory cells and those of the primary response is the subject of debate [83], and many theoretical formulations. Stromberg and Antia [33] suggested a quick way to mathematically simulate reinfection, using the same model structure, but assuming that the first immune cell compartment represents pre-existent memory (instead of naive precursors), initialized at the level of memory cells (*M*_*final*_) obtained or remaining from the primary response. Upon new pathogen encounter, these cells would then acquire effector function and drive rapid pathogen clearance, boosting adaptive immunity. Another way of extending the current model to accomodate secondary infection and the action of immune memory would be to modify the equations by adding a direct contribution into the *E* compartment by *M* cells, as a function of their stimulation by antigen (see S1 Text, part III). In the time scale considered in this paper, such conversion process (*M* → *E*) is broadly inconsequential for overall clearance dynamics. Our main results, highlighting the treatment-immunity interplay during primary infection, remain robust to this assumption. Yet, when simulating scenarios of secondary vs. primary infection (S10 Fig), the biological importance of pre-existing memory becomes clear, in particular the trade-off between host immunization and pathology reduction induced by antibiotic treatment during primary infection. More detailed conversion processes between effector and memory cell compartments could of course be modelled depending on data availability and the precise question.

In future studies, critical attention must be devoted to the optimality targets for treatment. If quantities to be optimized differ across settings, then optimal treatments will vary and may be difficult to compare in a standard manner. We show that different infection features have different sensitivity to treatment parameters. Thus, it is important to inform these control targets in a bottom-up fashion from clinical and medical considerations, but also from the broader epidemiological context of specific pathogens. For example, what is the order of priority for optimizing different health indicators? How does the symptom threshold, when patients seek specialized help, relate to the host immunity threshold? What is the upper bound for pathogen load within individual hosts to prevent onward transmission? What is a tolerable range for immunopathology? All these questions require integrative approaches at the medical-computational biology-and-immunology interface. Ideally, these should be achieved through the analysis of resistance evolution *in vivo*, empirical data from clinical outcomes of specific diseases, and integration with theoretical models.

Along similar lines, caution must be taken regarding the literal interpretation of the drug doses used in our model, reflecting more what bacteria ‘experience’. The translation of the modelled antibiotic dose to clinical prescriptions requires pharmacodynamic considerations [84]. A constant level of drug concentration, as the one assumed here, is unrealistic for real infection, except for cases of intravenous administration [85]. Yet, the behaviour described by our model, and the suggested trends, regarding the critical dose range and the susceptibility spectrum of co-infecting bacteria, are expected to be maintained, regardless of the corresponding real drug concentrations. Calibration of the model structure and parameters to real data from *in vivo*, *in vitro*, and clinical studies is a natural next step for validation.

Matching the *in-vitro* susceptibility of the pathogen and clinical prescription has been shown to be an important prognostic factor in real clinical settings [17, 86]. Our findings point to yet another dimension: accounting for the quantitative contribution of host immune responses, whether in immune competent or immunocompromised state. Depending on the type of infection, and host status, the optimal treatment parameters may vary. For some infections, such as *Staphylococcus aureus* bacteraemia or enterococcal endocarditis, prolonged treatment is recommended to prevent relapse [87]. Conversely, in other situations including otitis in children [88], the treatment of gonorrhoea [89], uncomplicated urinary tract infections in women [90], and uncomplicated cases of community acquired pneumonia [61], the roles for short courses of antibiotics appear well established.

We restricted our analysis to antigen-dependent immunity (type I), as this is likely to be most vulnerable to interference by the antibiotic treatment. Action of antigen-independent immunity (type II) could be included in our model as a reduction in net growth rate of bacteria within host (e.g. via a constant factor, or a function of time [31]). Alternatively, to account for the initial trigger by pathogen density, followed by programmed lymphocyte division, model extensions like those in [33, 37] could be adopted. In principle, programmed immune defenses, when added to the system, should weaken the competition between the drug and the other immune responses that are coupled to pathogen density, facilitating infection clearance, especially in scenarios of antibiotic-driven relapses. When only programmed immune defenses are available instead, then optimal therapies should approach aggressive treatment scenarios (Eq 19), where the interaction with the drug would occur only in one direction, from immunity to the drug, possibly through a time-dependent net growth rate of the pathogen.

As our understanding of pathogen population dynamics within host increases, by incorporating in higher quantitative detail the action of the immune system, a more promising and sustainable line of personalized antimicrobial therapy can be foreseen: the one based on a synergy between antimicrobial drugs and host immunity, whenever possible. In the future it will be important to robustly validate the sensitivity of treatment dynamics to pathogen and host parameters, initial conditions at treatment onset, and concentration of antibiotics. By emphasizing the dependence of therapeutic success on these crucial quantities, across classical and adaptive treatment regimes, our study calls for more empirical attention to host’s natural defenses in fighting drug-resistant infections.

## Supporting Information

S1 Text I.Model analysis: Equilibria and stability. II. Contraction phase of the immune response after adaptive treatment. III. Extending the model to represent secondary infection. (PDF)

S1 FigEffects of antibiotic therapy as a function of the time of treatment onset.A) *c* = 2.2 (high cost of resistance, as in Table 1). B) *c* = 0.1 (small cost of resistance). Other parameters as in Table 1. Infections were simulated for 30 days. The different lines depict the proportional reduction (on a log-scale) in different infection measures, relative to no-treatment: *M*(*D*) refers to final immune memory, *H*_*tot*_ to cumulative immunopathology, and *R*_*tot*_ to overall resistance. We see a maximum effect at intermediate delays for moderate doses below and within the critical range $\left[A_{m}^{*},A_{m}^{**}\right]$, signalling the benefit of host immunity involvement in clearance. This benefit does not apply when aggressive doses are deployed.(PDF)

S2 Fig Dose-duration interaction, for 2 treatment delays. A) *τ*_1_ = 2 days. B) *τ*_1_ = 4 days. Parameters as in Table 1. Top panel shows log-10 *R*_*tot*_ and bottom panel shows log-10 *H*_*tot*_, where infections were simulated for 30 days. The same outcomes can be obtained by trading off dose and treatment duration. When treatment onset exceeds the time required for immune stimulation (B), lower dose-duration (*A*_*m*_, *τ*_2_) combinations become more effective.
(PDF)

S3 Fig Dose-delay interaction determines multiple infection outcomes. A) *a* = 0.1 (high resistance of *B*_*r*_). B) *a* = 0.2 (lower resistance of *B*_*r*_). Parameters as in Table 1. Top panel shows log-ratios *R*_*tot*_ between treated and untreated infection, and bottom panel shows log-ratio *H*_*tot*_ between treated and untreated infection, where infections were simulated for 30 days. Negative values imply an improvement relative to no-treatment.
(PDF)

S4 Fig Resistance selection window for Fig 5 in the paper, related to classical treatment. We plot more clearly the range of dose-delay treatment combinations that lead to selection of the resistant sub-population, relative to an untreated infection. All parameters as specified in Fig 5 of the paper.
(PDF)

S5 FigTreatment duration and time to clearance for Fig 5 in the paper, related to classical treatment.We show a contourplot of the duration of treated infection across a range of dose-delay treatment combinations, for the case of *a* = 0.1. The cost of resistance is: A)-B) *c* = 2.2, C)-D) *c* = 1, E)-F) *c* = 0.1. The MSW is given by the region confined within the white dashed line. All parameters as specified in Fig 5 of the paper. The two columns correspond to the first and third column in Fig 5 respectively for treatment duration of 7 days and 15 days. Above the MSW, treatment duration reduces time to clearance for the same dose-delay combinations. Below the MSW, increasing treatment duration has no major effect. Notice that increasing treatment duration around the critical inhibitory dose for *B*_*r*_, namely around $A_{m}^{**}=\frac{r_{1}}{a\delta_{0}}$ can worsen treatment outcomes, inducing oscillatory dynamics (infection still persisting after 30 days).(PDF)

S6 FigOptimal classical treatments and non-waning immunity, *h* = 0.We show the resistance selection over treated infections, for different combinations of dose *A*_*m*_ and delay *τ*_1_ in the classical regime. The simulated values of *A*_*m*_ correspond to 30 doses in the range $\left[0.1A_{m}^{*},2A_{m}^{**}\right]$. The proportional change in the resistance burden over treated infection *R*_*tot*_ is measured relative to the one in untreated infection, as a function of antibiotic dose and timing of treatment onset for the same range of treatment scenarios as in Fig 5 of the paper. The resistance selection window is defined by the dashed white line (contour line corresponding to a proportional change of 1). Superimposed are treatment combinations that maintain infection duration within a factor of 1.1 relative to no treatment (yellow dots), and those that, in addition, satisfy a reduction in immunopathology by at least 2-logs order of magnitude (red dots). All parameters as in Table 1, unless otherwise stated. We vary the growth rate of the resistant bacteria to reflect different costs of resistance *c* = *r*_0_ − *r*_1_ (different rows). Duration of classical treatment is fixed to 7 days, except in C, F, I. We see again that moderate dose-delay combinations, applied below MSW at each infection, can be effective in clearing the pathogen, in synergy with host immune responses. The aggressive high doses instead are effective without relying on the contribution by the immune system.(PDF)

S7 Fig Immunity growing during adaptive treatment over a range of Ω and *σ*. We plot how total immunity changes during simulations of adaptive treatment with Ω > *k*, (blue lines) and superimpose the dynamics approximated by Eq 20 in the paper, in red. There is a clear match between the two, confirming the validity of this approximation. The range for Ω is [10^5^, 10^6^], while the rate of immune growth *σ* varies in [2, 4]. Parameters as in Table 1.
(PDF)

S8 Fig The duration of adaptive treatment(days) over a range of scenarios. A) Simulated. B) Theoretical expectation from Eq 21 in the paper. When strength of host immunity increases, clearance by adaptive treatment can be achieved starting at lower symptom thresholds. Parameters as in Table 1. The dose used is the minimum required to stop growth of the dominant sub-population at treatment onset (section 3.5 of paper), in this case drug-sensitive bacteria, because the assumed cost of resistance is high (*c* = 2.2). A similar plot can be obtained when the cost of resistance is lower, but in that case, the minimal inhibitory dose would be higher *A*_*m*_ = [*r*_1_ − *dI*(*t*_Ω_)]/(*aδ*_0_). The estimated duration of adaptive treatment will be insensitive to changes in *c*, as long as the right dose for no-growth is used.
(PDF)

S9 Fig Comparison of classical and adaptive regimes, for lower cost of resistance (*c* = 0.1). Analogous to Fig 9 of the paper. Different dose-delay treatment scenarios are simulated up to *T* = 30 days. In the classical regime, treatment duration is 7 days, while in the adaptive regime, drug uptake is related to bacterial density above the threshold Ω = *B*(*τ*_1_). Parameters as in Table 1, except for *r*_1_. A) Clearance of infection by 30 days can be obtained: via classical treatment only (red), via adaptive treatment only (green) or using either regime (blue). B) The amount of drug deployed in each treatment and the associated time to clearance. C) Treatment outcomes in those cases where both regimes clear infection (mean ± sd). D) Treatment outcomes when only one of the two regimes yields clearance (mean ± sd). Compared to Fig 9 of the paper, here, adaptive treatment achieves clearance also at sub-optimal delays, because for the same doses, growth during treatment of resistant bacteria, stimulates more immunity.
(PDF)

S10 Fig Secondary infection scenarios with *M* → *E* activation added to the basic model. Top row depicts primary infection in untreated and treated hosts. All parameters as in Table 1. *B*_*s*_ is depicted in blue, *B*_*r*_ in dashed red line, and *I* in thin black line. Treatment parameters are: *A*_*m*_ = 32 and *A*_*m*_ = 3 in the classical aggressive and moderate case, respectively with treatment duration of 7 days; and *A*_*m*_ = (*r*_0_ − *dI*)/*δ*_0_ in the adaptive regime. Dynamics after primary infection are run until *T* = 30 at which point a second infection is initiated with the same *B*(0) (Rows 2–4). The level of immune cells across all compartments at that time point is used to initialize the values for reinfection dynamics. Primary infection dynamics are robust to inclusion/omission of *M* → *E* activation. *M* → *E* conversion has major importance for subsequent infections. Rows 2–4 depict secondary infection of the same host for different parameters of immune memory activation. As immune memory activation in an already immunized host becomes stronger and more efficient, the pathogen load experienced by the host during a second infection is reduced. At such low densities, and perhaps more generally during secondary response, additional immune mechanisms may enhance pathogen control and facilitate clearance.
(PDF)
