# Supplementary material for: Integrating Antimicrobial Therapy with Host Immunity to Fight Drug-Resistant Infections: Classical vs. Adaptive Treatment
Source: PLoS Comput Biol. 2016 Apr 14;12(4):e1004857. doi: 10.1371/journal.pcbi.1004857 (PMC4831758; doi:10.1371/journal.pcbi.1004857)
Supplement: S1 Text — (PDF) [file pcbi.1004857.s001.pdf]

Supporting Information, S1 text

Related to the article: *Integrating antimicrobial therapy with host immunity to fight drug-resistant infections: classical vs. adaptive treatment*,
by Erida Gjini and Patricia H. Brito (PLoS Computational Biology 2016)

I. Model analysis: Equilibria and stability

Performing linear stability analysis, we find that the system admits a multitude of possible clearance equilibria, with the broad pattern that if there is sufficient immunity in terms of either precursor cell abundance or memory cells ($M^* + N^*$), the clearance steady-state with no pathogen and no effector cells ($B_s^* = B_r^* = 0, E^* = 0$) is neutrally stable. Typically, with $r_0 \geq r_1$, sufficient immunity means: $M^* + N^* > \frac{r_0}{d}$ (strictly greater), independently of how the total sum is partitioned among precursor and memory cells (since they both act to kill the pathogen). Typically in the absence of treatment, infection dynamics tend to this pathogen-free equilibrium. Notice that given the constraint of a finite time horizon T for our simulations, and the implementation of an extinction threshold when $B_s, B_r < B_{ext}$ in our simulated infections, the final level of immunity accumulated may be below this theoretical equilibrium value: $M^* = \lim_{t\to\infty} M(t)$.

In addition, there are two cases of chronic pathogen *persistence* within host at a non-trivial equilibrium:

i) when $M^* = r_1/d$ (exactly equal), and suboptimal immune memory is just sufficient to prevent growth of the drug-resistant subpopulation, whose persistence may be prolonged indefinitely ($B_r^* > 0, B_s^* = 0, E^* = 0, N^* = 0$). Inspecting of the corresponding eigenvalues of the Jacobian matrix evaluated at this equilibrium, we find that this steady state is unstable when $B_r^* > hk/\sigma$, and neutrally stable otherwise.

ii) when $M^* = r_0/d$, and the persistence of drug-sensitive bacteria is observed instead ($B_s^* > 0, B_r^* = 0, E^* = 0, N^* = 0$). The stability of this equilibrium requires $B_s^* \leq hk/\sigma$. Which one of these two occurs depends on how r_0 compares to r_1. If $r_0 \geq r_1$, then B_s persistence will be observed in the absence of the antibiotic, in this particular critical case.

Treatment interference

What may happen with treatment, is that due to the interference with immunity, the total immunity at the end of treatment may just about linger around this critical value, sufficient to halt pathogen growth. Subsequently, due to its coupling with waning immunity, the total pathogen population may begin to display oscillations around hk/σ, a value sufficient to make $dI/dt = 0$. Notice that if this immunity consisted only of persistent memory cells, there would not be potential for immune decay and oscillations: these occur because well into the course of infection, $I \approx E + M$, with $N \approx 0$ and M small, and effector cells prompt oscillatory behaviour, similar to predator-prey cycles.

Due to the advantage of the resistant subpopulation in the presence of the antibiotic ($a < 1$), the fitness differential between the two types may be reversed at high doses, leading to clearance of the drug-sensitive sub-population, but oscillatory persistence of B_r post-treatment.

II. Contraction phase of the immune response after adaptive treatment

The secondary phase of the immune response (when $I(0) \geq r_0/d$ and $B(0) \geq k$) can be approximated by the sub-system:

$$\frac{dB}{dt} \approx r_0 B - dBI \tag{1}$$

$$\frac{dI}{dt} \approx \Big(\sigma + h(1-f)\Big) I \frac{B}{k+B} - h(1-f)I \tag{2}$$

where $I = N + E + M$ and $B = B_s + B_r$. Dividing the two equations, integrating and re-arranging gives:

$$\Big(\sigma + h(1-f)\Big)\log\left(\frac{B+k}{B_0+k}\right) - h(1-f)\log\left(\frac{B}{B_0}\right) = r_0\log\left(\frac{I}{I_0}\right) - d(I - I_0). \tag{3}$$

This equation gives the relationship between the total number of immune cells, I, and parasite density, $B = B_s + B_r$, at any given time during the final immune growth and contraction phase, post-treatment, where $I_0 = I(0)$ and $B_0 = B(0)$. Thus, it allows us to calculate the level of immunity as a function of current pathogen load, and viceversa. At the end of adaptive treatment, the immune level is initially $I_0 = r_0/d$, and pathogen load initially is at the symptom threshold $B_0 = \Omega$. To find out what the value of total pathogen load is, when $I(t)$ hits $I_{crit} = r_0/d$ again during the decay phase, we just have to plug-in the above values in the above equation and solve for B. Since $I = I_0$, the right-hand-side of the equation becomes zero, and we obtain:

$$\Big(\sigma + h(1-f)\Big)\log\left(\frac{B+k}{\Omega+k}\right) = h(1-f)\log\left(\frac{B}{\Omega}\right), \tag{4}$$

which is equivalent to Eq.22 in the paper. Thus, if the solution of this equation with respect to B, sits below the extinction threshold, B_{ext}, clearance is guaranteed after adaptive treatment, otherwise oscillatory dynamics is induced between pathogen load peaking at Ω, and host immunity around r_0/d.

III. Extending the model to represent secondary infection

A possible model extension to represent secondary infection by the same pathogen could be to include an activation of pre-existent memory cells to re-stimulate effector cells, which then combat infection. Assuming the same functional response for this activation as a function of pathogen load, the model would be very similar to the baseline model presented in the paper, with only one addition in the dE/dt equation:

$$\frac{dB_s}{dt} = r_0 B_s - dB_s I - \delta_0 B_s \eta(t) A_m \quad (5)$$

$$\frac{dB_r}{dt} = r_1 B_r - dB_r I - \delta_1 B_r \eta(t) A_m \quad (6)$$

$$\frac{dN}{dt} = -\sigma N \frac{B}{k+B} \quad (7)$$

$$\frac{dE}{dt} = (2\sigma N + \sigma E)\frac{B}{k+B} - hE\left(1 - \frac{B}{k+B}\right) + \sigma_M M \frac{B}{k_M + B} \quad (8)$$

$$\frac{dM}{dt} = fEh\left(1 - \frac{B}{k+B}\right), \quad (9)$$

where $B(t) = B_s(t) + B_r(t)$ is the total pathogen load at time t, and $I(t) = N(t) + E(t) + M(t)$ is the total number of immune cells activated to clear the pathogen. In this model, since the basic motivation is to study secondary infection, the initial conditions representing this situation are likely $M(0) \gg N(0) > 0$, $B(0) > 0$ and $E(0) = 0$. The initial level of memory cells can be equal to the memory accumulated over the previous infection, or slightly lower, in case of memory decay in the meantime.

The parameters governing recruitment of memory cells into effector function are the rate of activation σ_M, and the half-saturation constant for antigen stimulation of memory cells k_M. These can be different from the original σ and k in the primary infection. For example, to represent rapid conversion of memory to effector cells we can assume that $\sigma_M > \sigma$, and to represent higher sensitivity to invading pathogen we may assume $k_M < k$. We do not use this model in the paper, although the dynamics for primary infection under this addition would remain largely unchanged. However we propose this as a possible model extension to study the importance of host immunization and sequential infection events.

In Figure S10, we illustrate hypothetical scenarios of secondary infection vs. primary infection under this model. We observe that the main features of the dynamics of pri-

mary infection remain robust (top panel of Figure S10). Furthermore, the biological role of pre-existing memory in secondary infection becomes clear: moderate treatment during primary exposure, leading to substantial host immunization, suppresses pathogen growth in subsequent exposures. In contrast, when aggressive treatments are applied in primary exposure, insufficient immune memory levels permit transient pathogen growth during secondary infections. In particular, the second row panel of Figure S10 illustrates that interference by treatment during primary exposure can lead to reinfections that resemble primary infection, where high pathogen loads would require further antibiotic treatment. If a more moderate regime would have been used instead, bacterial loads in secondary infections will be controlled by immunity. The immune dynamics during secondary response naturally depends on the exact parameters governing $M \to E$ activation. As expected, the faster and stronger this activation is (bottom three panels in Figure S10), the lower the pathogen load and the faster the clearance. These brief insights into secondary infection call for further modeling work in this direction in the future.
