# Supplementary material for: Integrating Antimicrobial Therapy with Host Immunity to Fight Drug-Resistant Infections: Classical vs. Adaptive Treatment
Source: PLoS Comput Biol. 2016 Apr 14;12(4):e1004857. doi: 10.1371/journal.pcbi.1004857 (PMC4831758; doi:10.1371/journal.pcbi.1004857)
Supplement: S1 Fig — A) c = 2.2 (high cost of resistance, as in Table 1). B) c = 0.1 (small cost of resistance). Other parameters as in Table 1. Infections were simulated for 30 days. The different lines depict the proportional reduction (on a log-scale) in different infection measures, relative to no-treatment: M(D) refers to final immune memory, Htot to cumulative immunopathology, and Rtot to overall resistance. We see a maximum effect at intermediate delays for moderate doses below and within the critical range [Am*,Am**], signalling the benefit of host immunity involvement in clearance. This benefit does not apply when aggressive doses are deployed. (PDF) [file pcbi.1004857.s002.pdf]

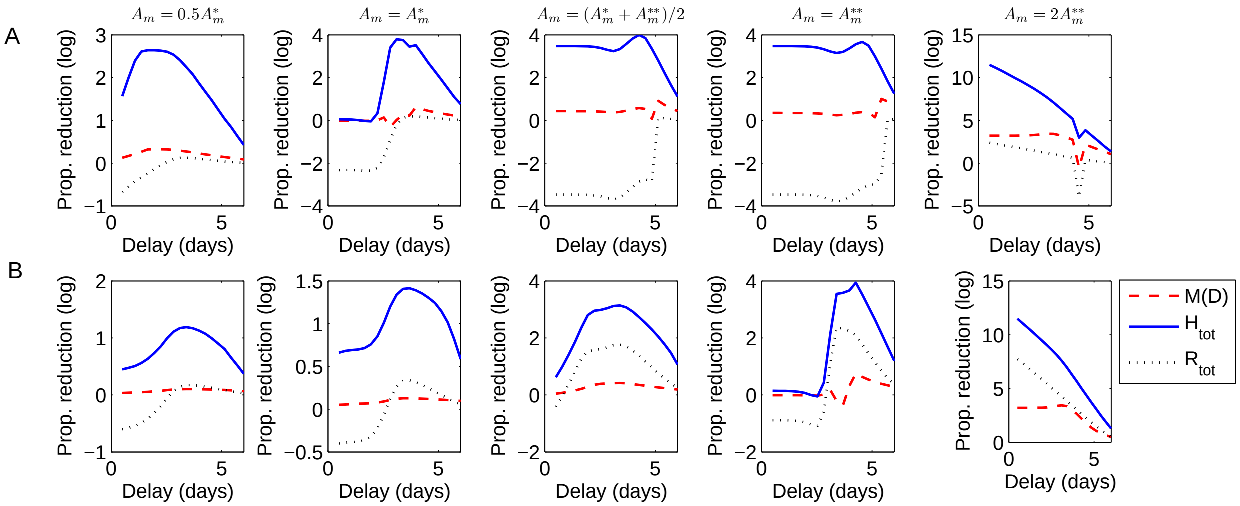

Figure S1: **Effects of antibiotic therapy as a function of the time of treatment onset.** A) $c = 2.2$ (high cost of resistance, as in Table **??**). B) $c = 0.1$ (small cost of resistance). Other parameters as in Table 1. Infections were simulated for 30 days. The different lines depict the proportional reduction (on a log-scale) in different infection measures, relative to no-treatment: $M(D)$ refers to final immune memory, H_{tot} to cumulative immunopathology, and R_{tot} to overall resistance. We see a maximum effect at intermediate delays for moderate doses below and within the critical range $[A_m^*, A_m^{**}]$, signalling the benefit of host immunity involvement in clearance. This benefit does not apply when aggressive doses are deployed.
