# Supplementary material for: Integrating Antimicrobial Therapy with Host Immunity to Fight Drug-Resistant Infections: Classical vs. Adaptive Treatment
Source: PLoS Comput Biol. 2016 Apr 14;12(4):e1004857. doi: 10.1371/journal.pcbi.1004857 (PMC4831758; doi:10.1371/journal.pcbi.1004857)
Supplement: S2 Fig — A) τ1 = 2 days. B) τ1 = 4 days. Parameters as in Table 1. Top panel shows log-10 Rtot and bottom panel shows log-10 Htot, where infections were simulated for 30 days. The same outcomes can be obtained by trading off dose and treatment duration. When treatment onset exceeds the time required for immune stimulation (B), lower dose-duration (Am, τ2) combinations become more effective. (PDF) [file pcbi.1004857.s003.pdf]

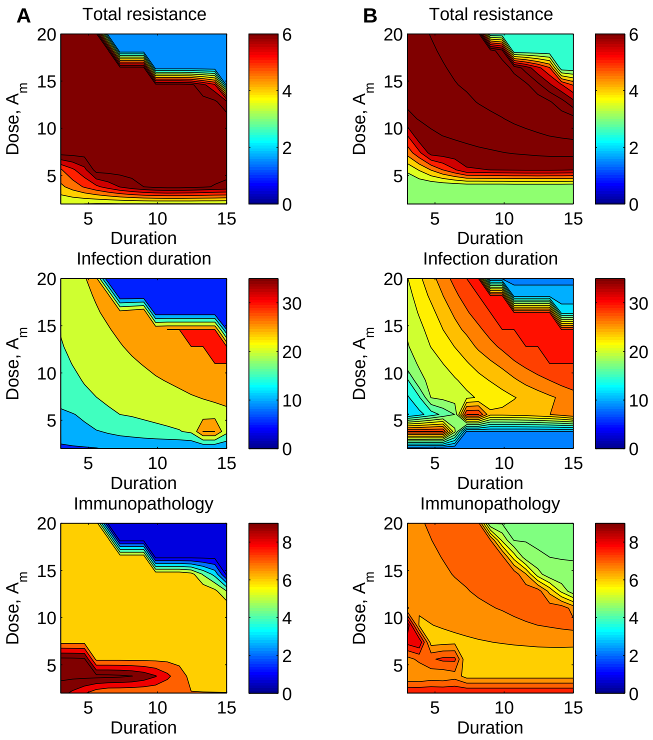

Figure S2: **Dose-duration interaction, for 2 treatment delays.** A) $\tau_1 = 2$ days. B) $\tau_1 = 4$ days. Parameters as in Table 1. Top panel shows log-10 R_{tot} and bottom panel shows log-10 H_{tot}, where infections were simulated for 30 days. The same outcomes can be obtained by trading off dose and treatment duration. When treatment onset exceeds the time required for immune stimulation (B), lower dose-duration (A_m, τ_2) combinations become more effective.
