# Supplementary material for: Integrating Antimicrobial Therapy with Host Immunity to Fight Drug-Resistant Infections: Classical vs. Adaptive Treatment
Source: PLoS Comput Biol. 2016 Apr 14;12(4):e1004857. doi: 10.1371/journal.pcbi.1004857 (PMC4831758; doi:10.1371/journal.pcbi.1004857)
Supplement: S3 Fig — A) a = 0.1 (high resistance of Br). B) a = 0.2 (lower resistance of Br). Parameters as in Table 1. Top panel shows log-ratios Rtot between treated and untreated infection, and bottom panel shows log-ratio Htot between treated and untreated infection, where infections were simulated for 30 days. Negative values imply an improvement relative to no-treatment. (PDF) [file pcbi.1004857.s004.pdf]

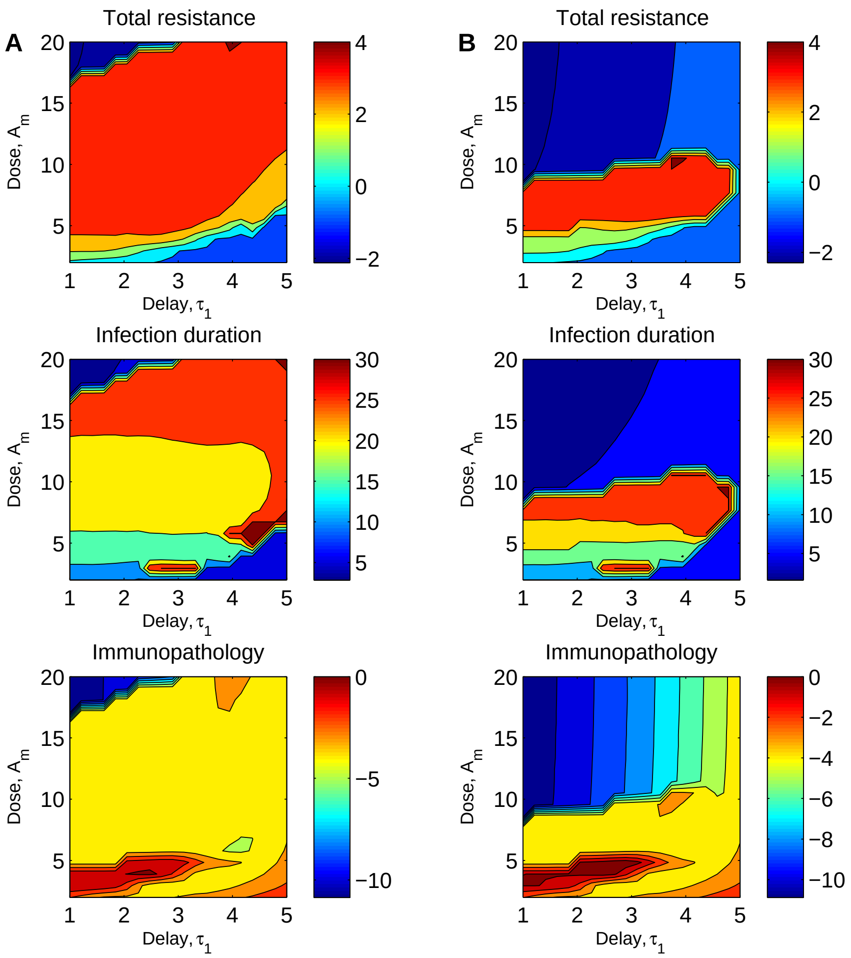

Figure S3: **Dose-delay interaction determines multiple infection outcomes**. A) $a = 0.1$ (high resistance of B_r). B) $a = 0.2$ (lower resistance of B_r). Parameters as in Table 1. Top panel shows log-ratios R_{tot} between treated and untreated infection, and bottom panel shows log-ratio H_{tot} between treated and untreated infection, where infections were simulated for 30 days. Negative values imply an improvement relative to no-treatment.
