# Supplementary material for: Integrating Antimicrobial Therapy with Host Immunity to Fight Drug-Resistant Infections: Classical vs. Adaptive Treatment
Source: PLoS Comput Biol. 2016 Apr 14;12(4):e1004857. doi: 10.1371/journal.pcbi.1004857 (PMC4831758; doi:10.1371/journal.pcbi.1004857)
Supplement: S4 Fig — We plot more clearly the range of dose-delay treatment combinations that lead to selection of the resistant sub-population, relative to an untreated infection. All parameters as specified in Fig 5 of the paper. (PDF) [file pcbi.1004857.s005.pdf]

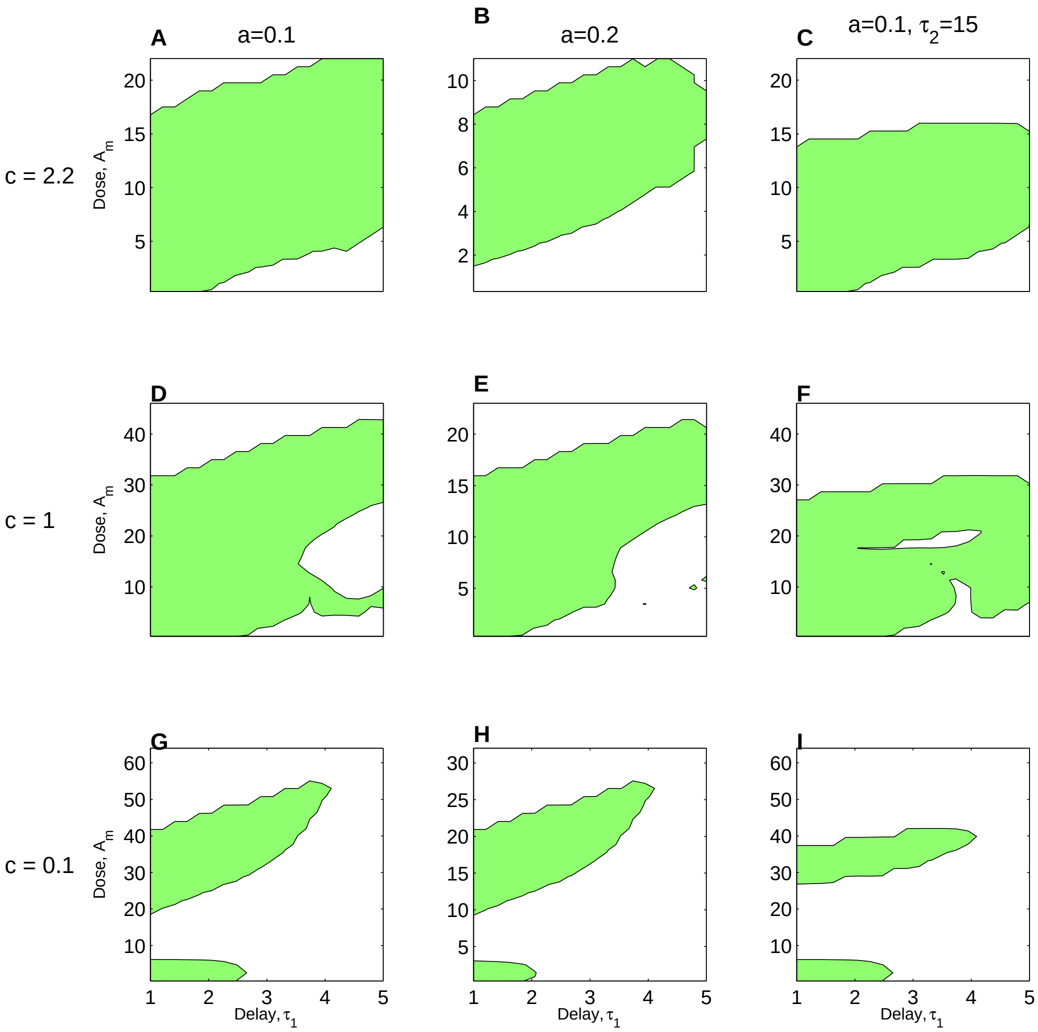

Figure S4: **Resistance selection window for Fig. 5 in the paper, related to classical treatment.** We plot more clearly the range of dose-delay treatment combinations that lead to selection of the resistant sub-population, relative to an untreated infection. All parameters as specified in Fig. 5 of the paper.
