# Supplementary material for: Integrating Antimicrobial Therapy with Host Immunity to Fight Drug-Resistant Infections: Classical vs. Adaptive Treatment
Source: PLoS Comput Biol. 2016 Apr 14;12(4):e1004857. doi: 10.1371/journal.pcbi.1004857 (PMC4831758; doi:10.1371/journal.pcbi.1004857)
Supplement: S5 Fig — We show a contourplot of the duration of treated infection across a range of dose-delay treatment combinations, for the case of a = 0.1. The cost of resistance is: A)-B) c = 2.2, C)-D) c = 1, E)-F) c = 0.1. The MSW is given by the region confined within the white dashed line. All parameters as specified in Fig 5 of the paper. The two columns correspond to the first and third column in Fig 5 respectively for treatment duration of 7 days and 15 days. Above the MSW, treatment duration reduces time to clearance for the same dose-delay combinations. Below the MSW, increasing treatment duration has no major effect. Notice that increasing treatment duration around the critical inhibitory dose for Br, namely around Am**=r1aδ0 can worsen treatment outcomes, inducing oscillatory dynamics (infection still persisting after 30 days). (PDF) [file pcbi.1004857.s006.pdf]

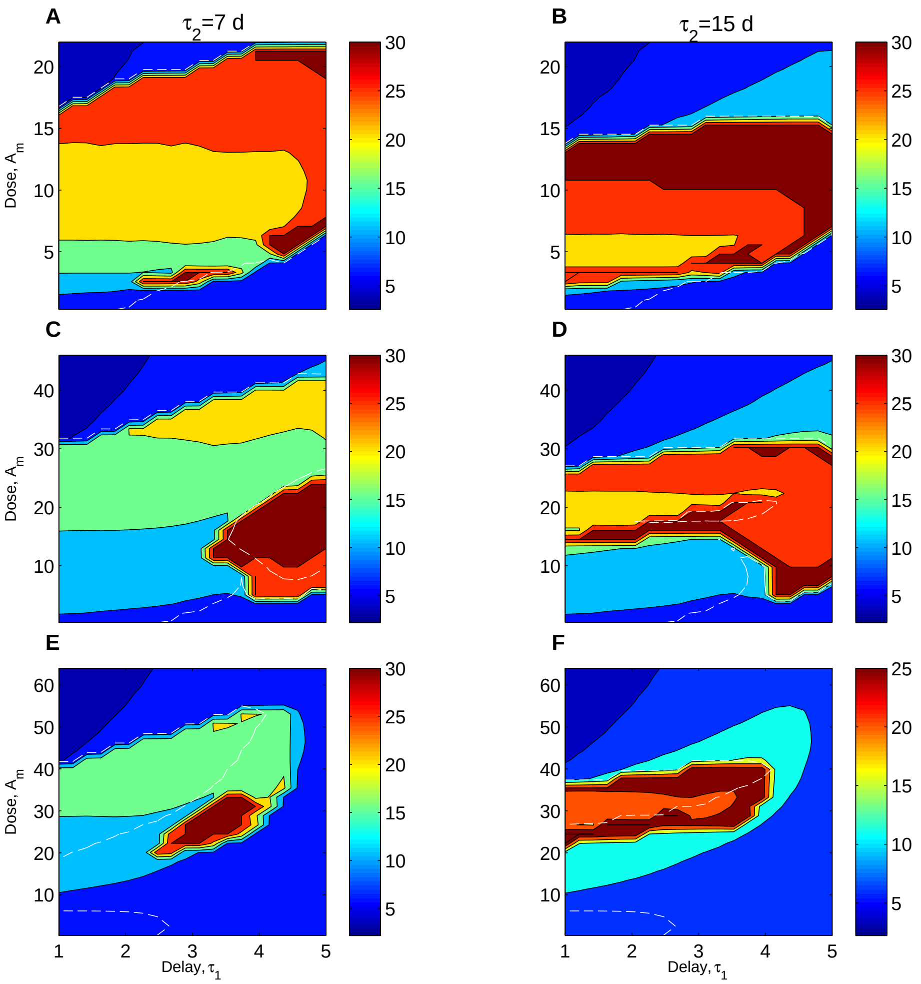


Figure S5: **Treatment duration and time to clearance for Fig. 5 in the paper, related to classical treatment.** We show a contourplot of the duration of treated infection across a range of dose-delay treatment combinations, for the case of $a = 0.1$. The cost of resistance is: A)-B) $c = 2.2$, C)-D) $c = 1$, E)-F) $c = 0.1$. The MSW is given by the region confined within the white dashed line. All parameters as specified in Fig. 5 of the paper. The two columns correspond to the first and third column in Fig. 5 respectively for treatment duration of 7 days and 15 days. Above the MSW, treatment duration reduces time to clearance for the same dose-delay combinations. Below the MSW, increasing treatment duration has no major effect. Notice that increasing treatment duration around the critical inhibitory dose for $B_r$, namely around $A_m^{**} = \frac{r_1}{a\delta_0}$ can worsen treatment outcomes, inducing oscillatory dynamics (infection still persisting after 30 days).
