# Supplementary material for: Integrating Antimicrobial Therapy with Host Immunity to Fight Drug-Resistant Infections: Classical vs. Adaptive Treatment
Source: PLoS Comput Biol. 2016 Apr 14;12(4):e1004857. doi: 10.1371/journal.pcbi.1004857 (PMC4831758; doi:10.1371/journal.pcbi.1004857)
Supplement: S6 Fig — We show the resistance selection over treated infections, for different combinations of dose Am and delay τ1 in the classical regime. The simulated values of Am correspond to 30 doses in the range [0.1Am*,2Am**]. The proportional change in the resistance burden over treated infection Rtot is measured relative to the one in untreated infection, as a function of antibiotic dose and timing of treatment onset for the same range of treatment scenarios as in Fig 5 of the paper. The resistance selection window is defined by the dashed white line (contour line corresponding to a proportional change of 1). Superimposed are treatment combinations that maintain infection duration within a factor of 1.1 relative to no treatment (yellow dots), and those that, in addition, satisfy a reduction in immunopathology by at least 2-logs order of magnitude (red dots). All parameters as in Table 1, unless otherwise stated. We vary the growth rate of the resistant bacteria to reflect different costs of resistance c = r0 − r1 (different rows). Duration of classical treatment is fixed to 7 days, except in C, F, I. We see again that moderate dose-delay combinations, applied below MSW at each infection, can be effective in clearing the pathogen, in synergy with host immune responses. The aggressive high doses instead are effective without relying on the contribution by the immune system. (PDF) [file pcbi.1004857.s007.pdf]

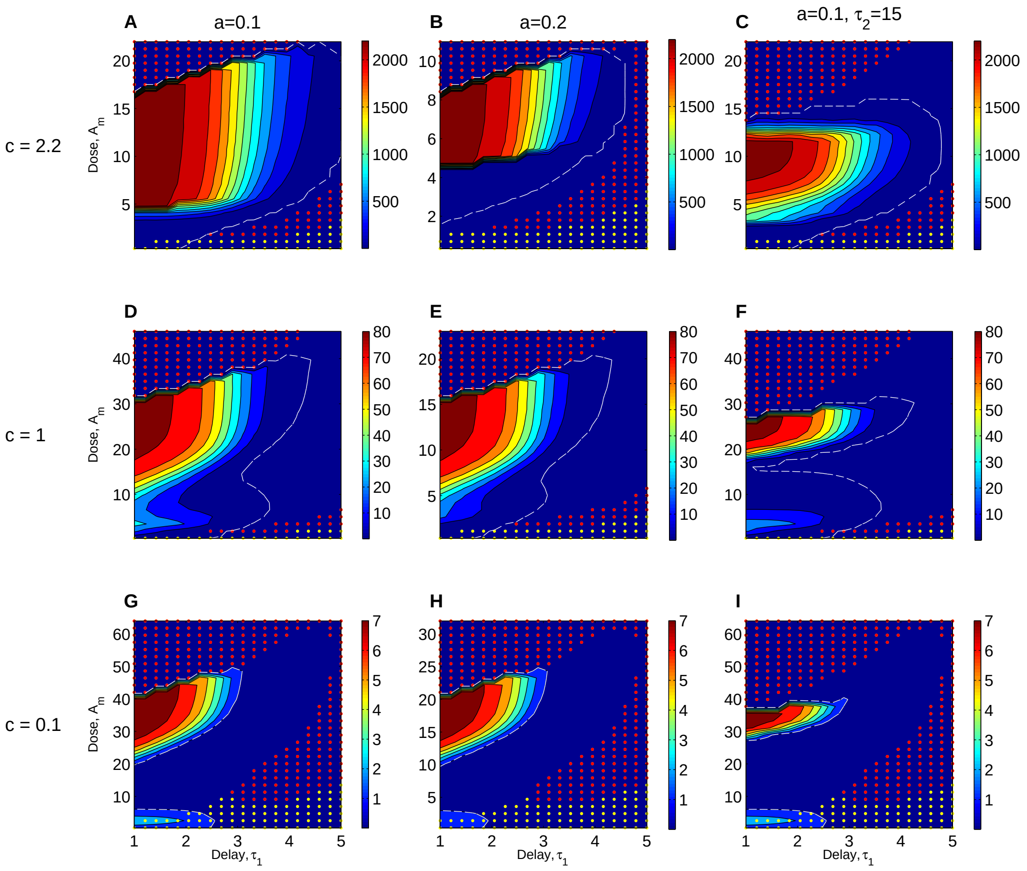


Figure S6: **Optimal classical treatments and non-waning immunity,** $h = 0$**.** We show the resistance selection over treated infections, for different combinations of dose $A_m$ and delay $\tau_1$ in the classical regime. The simulated values of $A_m$ correspond to 30 doses in the range $[0.1A^*_m, 2A^{**}_m]$. The proportional change in the resistance burden over treated infection $R_{tot}$ is measured relative to the one in untreated infection, as a function of antibiotic dose and timing of treatment onset for the same range of treatment scenarios as in Fig. 5 of the paper. The resistance selection window is defined by the dashed white line (contour line corresponding to a proportional change of 1). Superimposed are treatment combinations that maintain infection duration within a factor of 1.1 relative to no treatment (yellow dots), and those that, in addition, satisfy a reduction in immunopathology by at least 2-logs order of magnitude (red dots). All parameters as in Table 1, unless otherwise stated. We vary the growth rate of the resistant bacteria to reflect different costs of resistance $c = r_0 - r_1$ (different rows). Duration of classical treatment is fixed to 7 days, except in C,F,I. We see again that moderate dose-delay combinations, applied below MSW at each infection, can be effective in clearing the pathogen, in synergy with host immune responses. The aggressive high doses instead are effective without relying on the contribution by the immune system.
