# Supplementary material for: Integrating Antimicrobial Therapy with Host Immunity to Fight Drug-Resistant Infections: Classical vs. Adaptive Treatment
Source: PLoS Comput Biol. 2016 Apr 14;12(4):e1004857. doi: 10.1371/journal.pcbi.1004857 (PMC4831758; doi:10.1371/journal.pcbi.1004857)
Supplement: S7 Fig — We plot how total immunity changes during simulations of adaptive treatment with Ω > k, (blue lines) and superimpose the dynamics approximated by Eq 20 in the paper, in red. There is a clear match between the two, confirming the validity of this approximation. The range for Ω is [105, 106], while the rate of immune growth σ varies in [2, 4]. Parameters as in Table 1. (PDF) [file pcbi.1004857.s008.pdf]

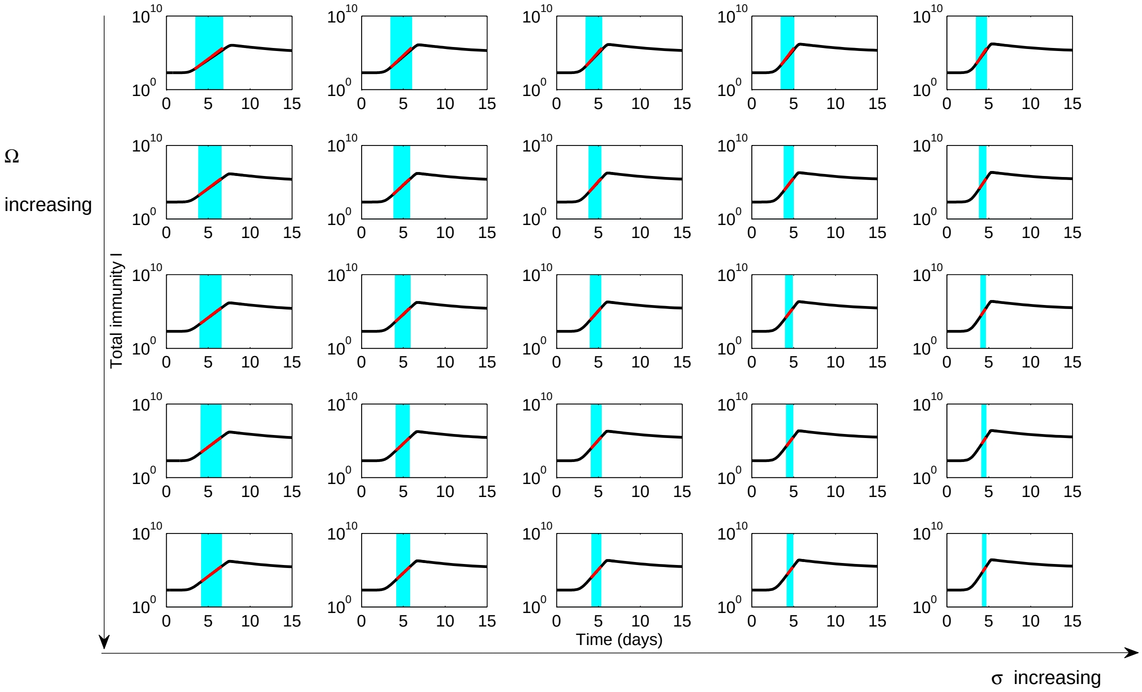

Figure S7: **Immunity growing during adaptive treatment over a range of Ω and σ.** We plot how total immunity changes during simulations of adaptive treatment with $\Omega > k$, (blue lines) and superimpose the dynamics approximated by Equation 20 in the paper, in red. There is a clear match between the two, confirming the validity of this approximation.The range for Ω is $[10^5, 10^6]$, while the rate of immune growth σ varies in [2,4]. Parameters as in Table 1.
