# Supplementary material for: Integrating Antimicrobial Therapy with Host Immunity to Fight Drug-Resistant Infections: Classical vs. Adaptive Treatment
Source: PLoS Comput Biol. 2016 Apr 14;12(4):e1004857. doi: 10.1371/journal.pcbi.1004857 (PMC4831758; doi:10.1371/journal.pcbi.1004857)
Supplement: S8 Fig — A) Simulated. B) Theoretical expectation from Eq 21 in the paper. When strength of host immunity increases, clearance by adaptive treatment can be achieved starting at lower symptom thresholds. Parameters as in Table 1. The dose used is the minimum required to stop growth of the dominant sub-population at treatment onset (section 3.5 of paper), in this case drug-sensitive bacteria, because the assumed cost of resistance is high (c = 2.2). A similar plot can be obtained when the cost of resistance is lower, but in that case, the minimal inhibitory dose would be higher Am = [r1 − dI(tΩ)]/(aδ0). The estimated duration of adaptive treatment will be insensitive to changes in c, as long as the right dose for no-growth is used. (PDF) [file pcbi.1004857.s009.pdf]

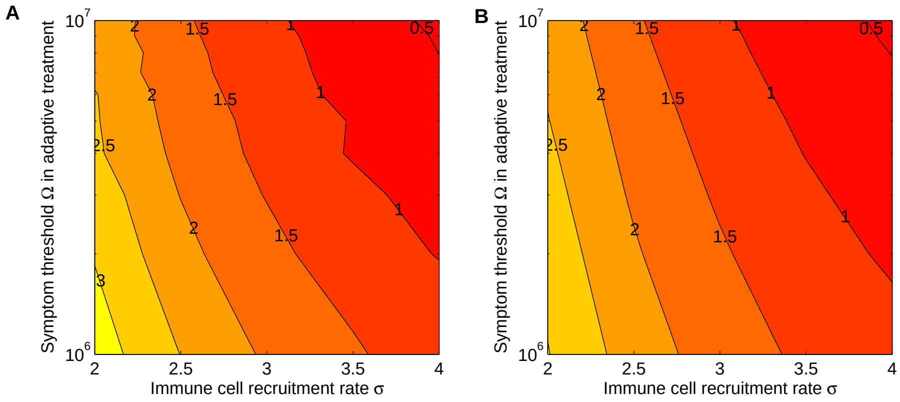

Figure S8: **The duration of adaptive treatment(days) over a range of scenarios.** A) Simulated. B) Theoretical expectation from Eq. 21 in the paper. When strength of host immunity increases, clearance by adaptive treatment can be achieved starting at lower symptom thresholds. Parameters as in Table 1. The dose used is the minimum required to stop growth of the dominant sub-population at treatment onset (section 3.5 of paper), in this case drug-sensitive bacteria, because the assumed cost of resistance is high ($c = 2.2$). A similar plot can be obtained when the cost of resistance is lower, but in that case, the minimal inhibitory dose would be higher $A_m = [r_1 - dI(t_\Omega)]/(a\delta_0)$. The estimated duration of adaptive treatment will be insensitive to changes in c, as long as the right dose for no-growth is used.
