# Supplementary material for: Integrating Antimicrobial Therapy with Host Immunity to Fight Drug-Resistant Infections: Classical vs. Adaptive Treatment
Source: PLoS Comput Biol. 2016 Apr 14;12(4):e1004857. doi: 10.1371/journal.pcbi.1004857 (PMC4831758; doi:10.1371/journal.pcbi.1004857)
Supplement: S9 Fig — Analogous to Fig 9 of the paper. Different dose-delay treatment scenarios are simulated up to T = 30 days. In the classical regime, treatment duration is 7 days, while in the adaptive regime, drug uptake is related to bacterial density above the threshold Ω = B(τ1). Parameters as in Table 1, except for r1. A) Clearance of infection by 30 days can be obtained: via classical treatment only (red), via adaptive treatment only (green) or using either regime (blue). B) The amount of drug deployed in each treatment and the associated time to clearance. C) Treatment outcomes in those cases where both regimes clear infection (mean ± sd). D) Treatment outcomes when only one of the two regimes yields clearance (mean ± sd). Compared to Fig 9 of the paper, here, adaptive treatment achieves clearance also at sub-optimal delays, because for the same doses, growth during treatment of resistant bacteria, stimulates more immunity. (PDF) [file pcbi.1004857.s010.pdf]

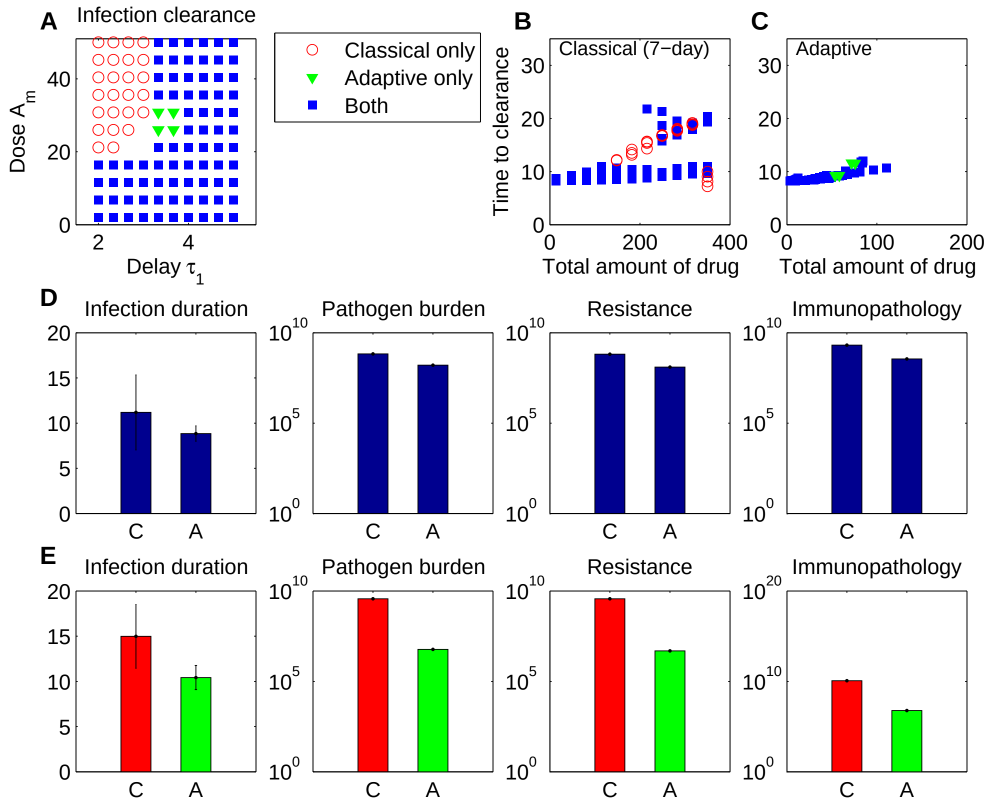

Figure S9: **Comparison of classical and adaptive regimes, for lower cost of resistance ($c = 0.1$).** Analogous to Fig. 9 of the paper. Different dose-delay treatment scenarios are simulated up to $T = 30$ days. In the classical regime, treatment duration is 7 days, while in the adaptive regime, drug uptake is related to bacterial density above the threshold $\Omega = B(\tau_1)$. Parameters as in Table 1, except for r_1. A) Clearance of infection by 30 days can be obtained: via classical treatment only (red), via adaptive treatment only (green) or using either regime (blue). B) The amount of drug deployed in each treatment and the associated time to clearance. C) Treatment outcomes in those cases where both regimes clear infection (mean ± sd). D) Treatment outcomes when only one of the two regimes yields clearance (mean ± sd). Compared to Fig. 9 of the paper, here, adaptive treatment achieves clearance also at sub-optimal delays, because for the same doses, growth during treatment of resistant bacteria, stimulates more immunity.
