# Supplementary material for: Integrating Antimicrobial Therapy with Host Immunity to Fight Drug-Resistant Infections: Classical vs. Adaptive Treatment
Source: PLoS Comput Biol. 2016 Apr 14;12(4):e1004857. doi: 10.1371/journal.pcbi.1004857 (PMC4831758; doi:10.1371/journal.pcbi.1004857)
Supplement: S10 Fig — Top row depicts primary infection in untreated and treated hosts. All parameters as in Table 1. Bs is depicted in blue, Br in dashed red line, and I in thin black line. Treatment parameters are: Am = 32 and Am = 3 in the classical aggressive and moderate case, respectively with treatment duration of 7 days; and Am = (r0 − dI)/δ0 in the adaptive regime. Dynamics after primary infection are run until T = 30 at which point a second infection is initiated with the same B(0) (Rows 2–4). The level of immune cells across all compartments at that time point is used to initialize the values for reinfection dynamics. Primary infection dynamics are robust to inclusion/omission of M → E activation. M → E conversion has major importance for subsequent infections. Rows 2–4 depict secondary infection of the same host for different parameters of immune memory activation. As immune memory activation in an already immunized host becomes stronger and more efficient, the pathogen load experienced by the host during a second infection is reduced. At such low densities, and perhaps more generally during secondary response, additional immune mechanisms may enhance pathogen control and facilitate clearance. (PDF) [file pcbi.1004857.s011.pdf]

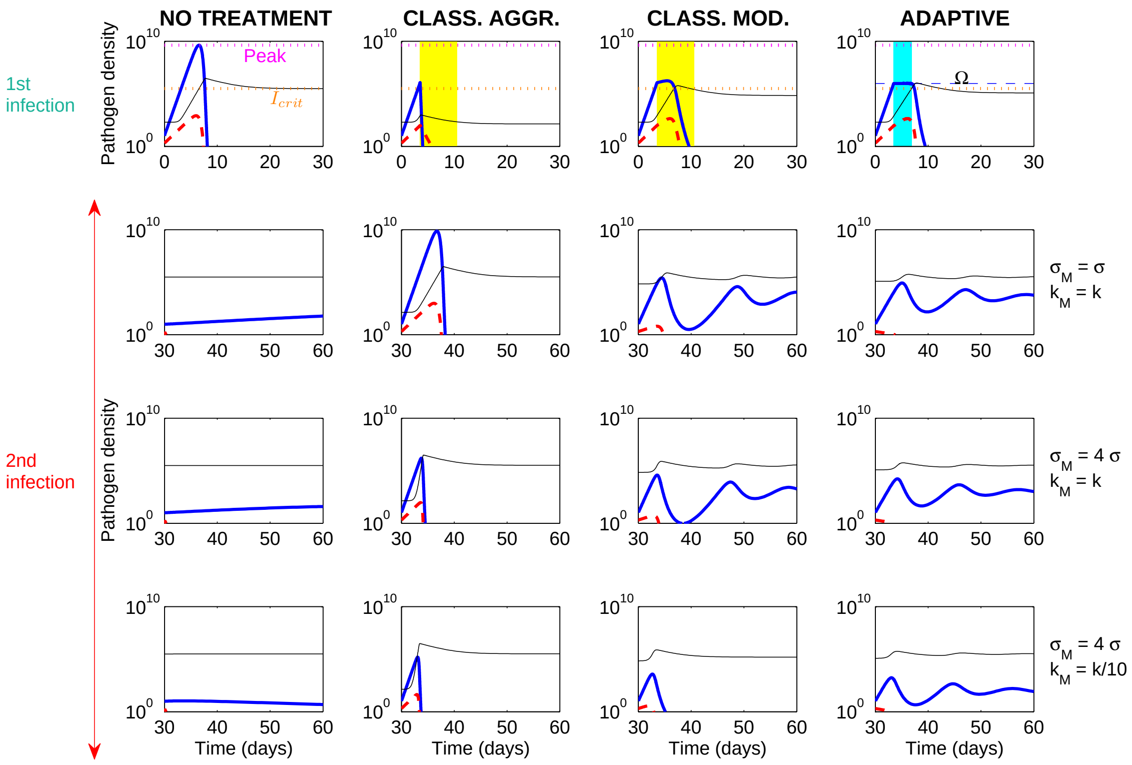

Figure S10: **Secondary infection scenarios with $M \rightarrow E$ activation added to the basic model.** Top row depicts primary infection in untreated and treated hosts. All parameters as in Table 1. B_s is depicted in blue, B_r in dashed red line, and I in thin black line. Treatment parameters are: $A_m = 32$ and $A_m = 3$ in the classical aggressive and moderate case, respectively with treatment duration of 7 days; and $A_m = (r_0 - dI)/\delta_0$ in the adaptive regime. Dynamics after primary infection are run until $T = 30$ at which point a second infection is initiated with the same $B(0)$ (Rows 2-4). The level of immune cells across all compartments at that time point is used to initialize the values for reinfection dynamics. Primary infection dynamics are robust to inclusion/omission of $M \rightarrow E$ activation. $M \rightarrow E$ conversion has major importance for subsequent infections. Rows 2-4 depict secondary infection of the same host for different parameters of immune memory activation. As immune memory activation in an already immunized host becomes stronger and more efficient, the pathogen load experienced by the host during a second infection is reduced. At such low densities, and perhaps more generally during secondary response, additional immune mechanisms may enhance pathogen control and facilitate clearance.
